# Supplementary material for: DOT1L inhibition attenuates graft-versus-host disease by allogeneic T cells in adoptive immunotherapy models
Source: Nat Commun. 2018 May 15;9:1915. doi: 10.1038/s41467-018-04262-0 (PMC5954061; doi:10.1038/s41467-018-04262-0)
Supplement: Supplementary file 1 — Supplementary Information [file 41467_2018_4262_MOESM1_ESM.pdf]

Supplementary Information

DOT1L inhibition attenuates graft-versus-host disease by allogeneic T cells in adoptive immunotherapy models

Kagoya et al.

Supplementary Figure 1

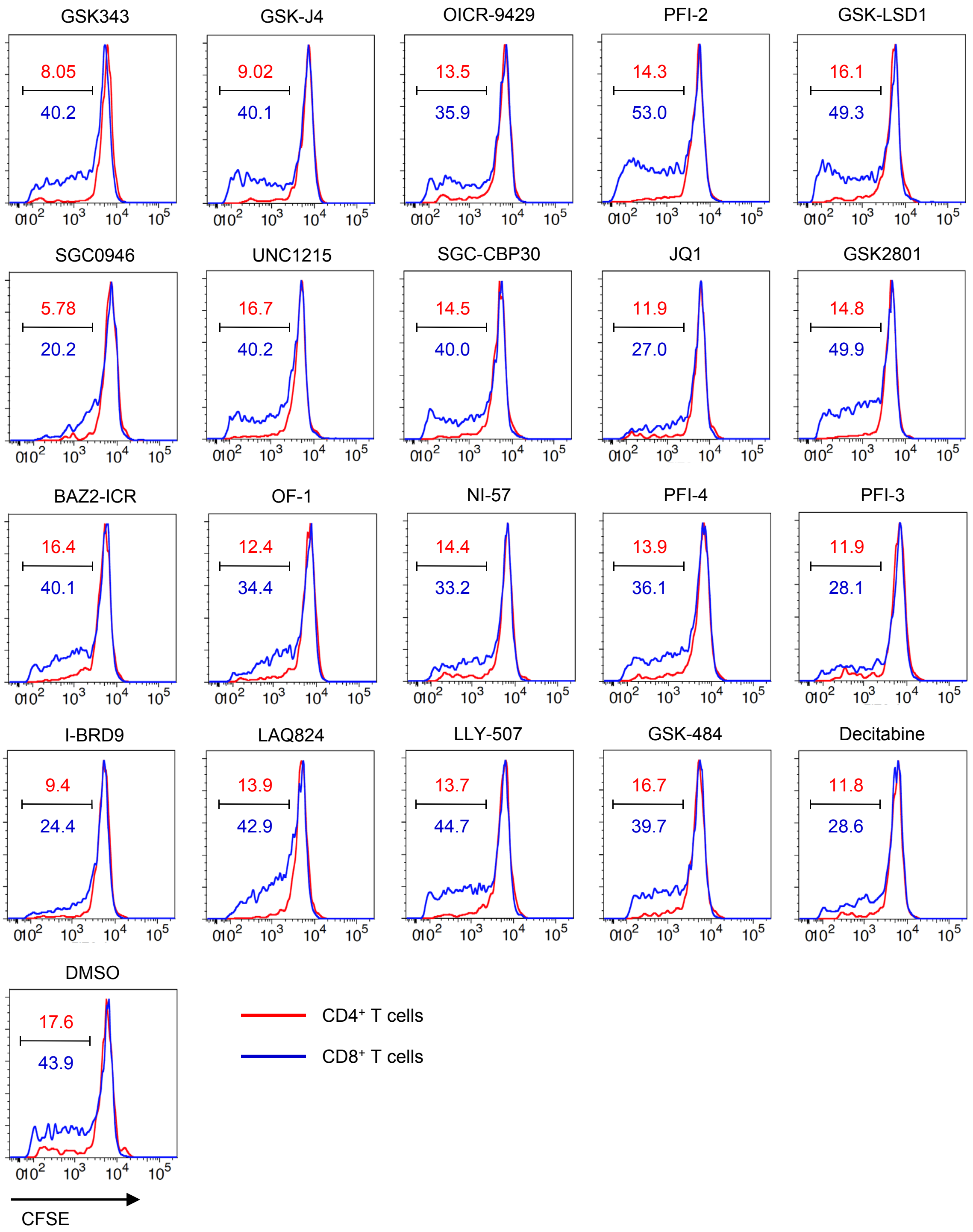

Supplementary Figure 1. Cell division of probe-treated T cells after coculture with allogeneic peripheral blood mononuclear cells (PBMC). $CD3^+$ T cells were stimulated with aAPC/mOKT3 and pretreated with individual chemical probes for 6 days. The T cells were then rested overnight, labeled with CFSE, and cocultured with allogeneic PBMC depleted of $CD3^+$ T cells for four days in the presence of each chemical probe. Representative CFSE flow cytometry plots for $CD4^+$ and $CD8^+$ T cells are shown (n=3 for each).

Supplementary Figure 2

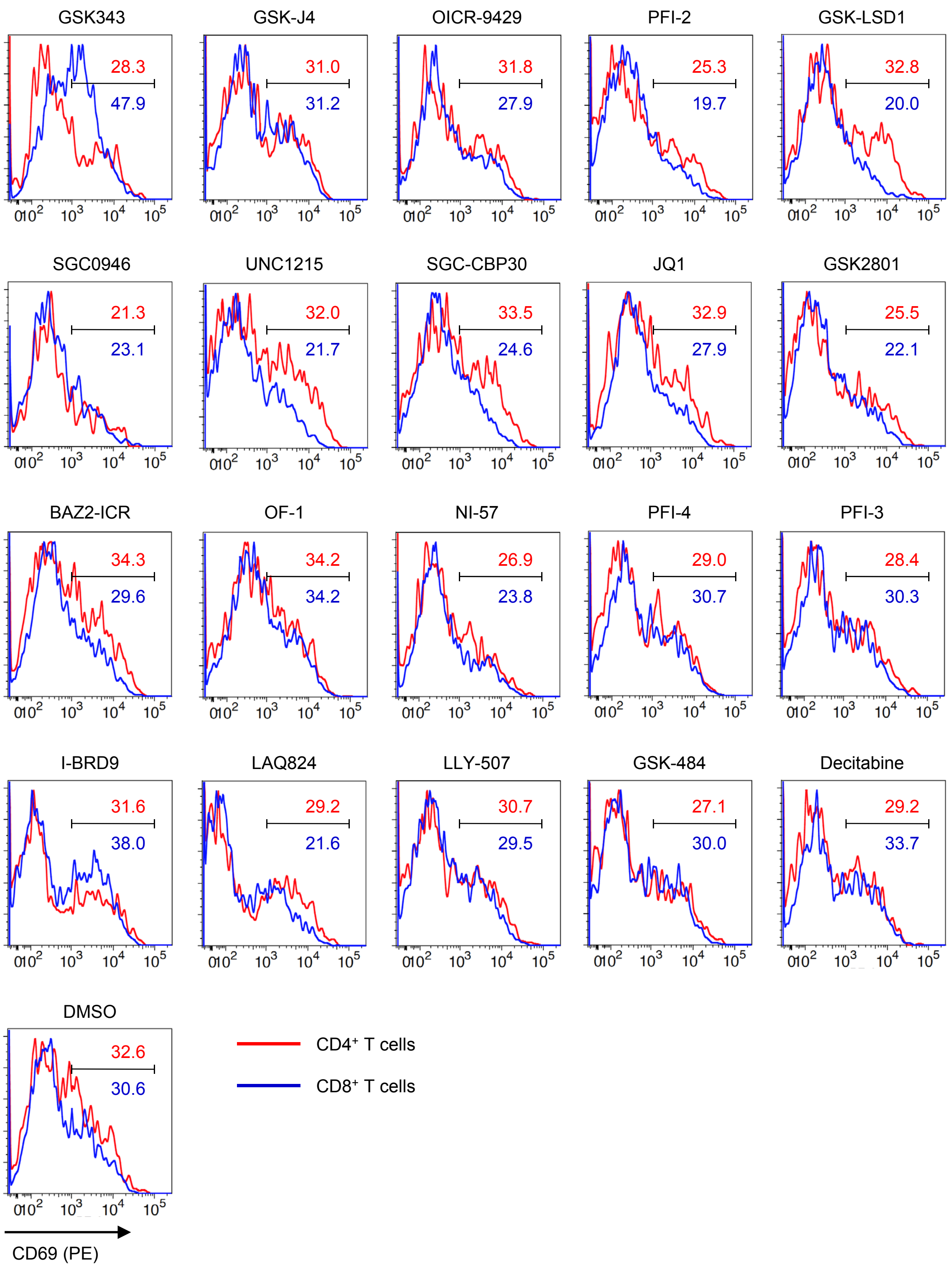

Supplementary Figure 2. Expression of CD69 in probe-treated T cells after coculture with allogeneic PBMC. CD3$^+$ T cells were cultured as described in Supplementary Fig. 1 and were analyzed for CD69 expression. Representative flow cytometry plots of CD69 expression in CD4$^+$ and CD8$^+$ T cells are shown (n=3 for each).

Supplementary Figure 3

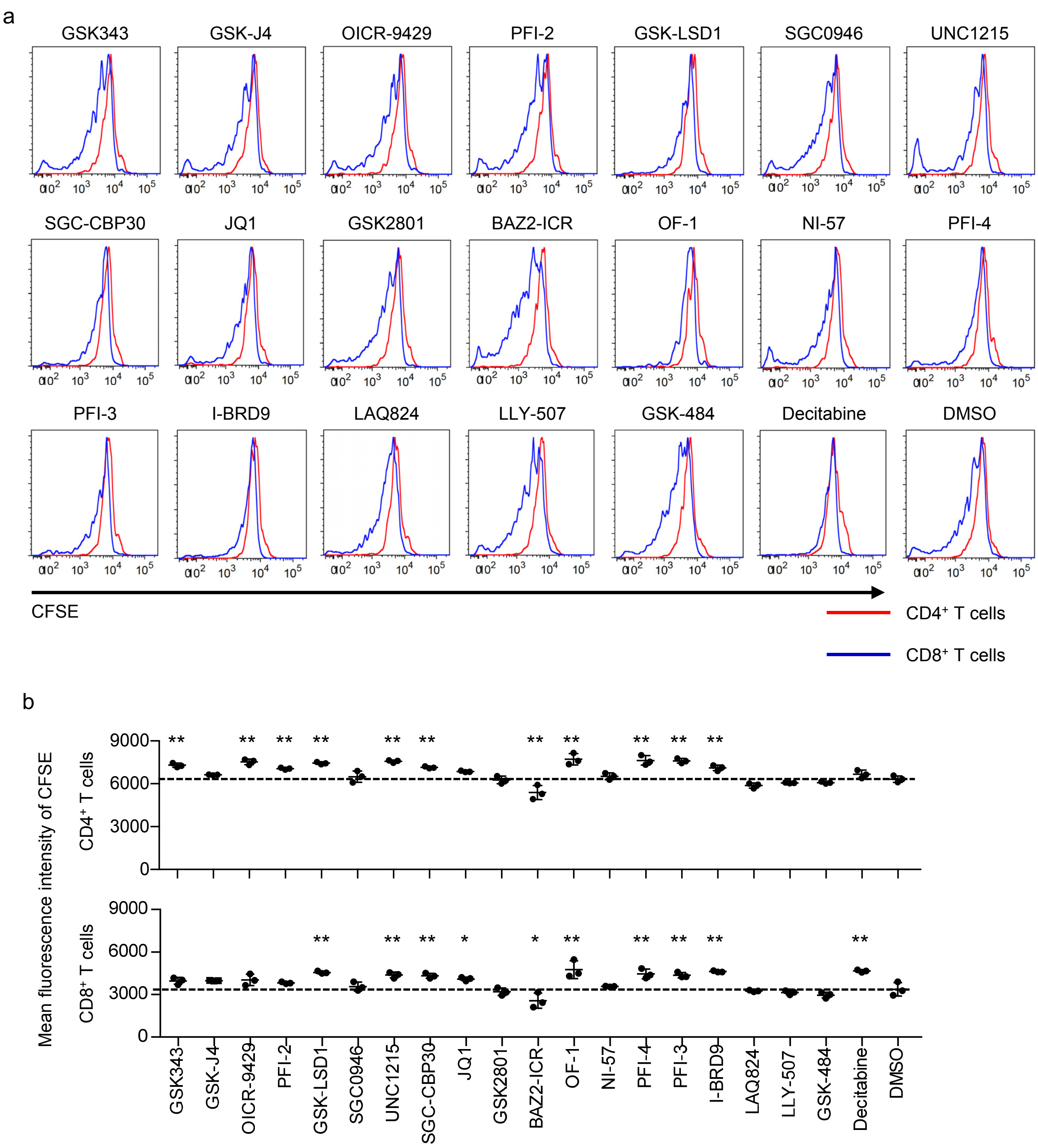

Supplementary Figure 3. Proliferation of probe-treated T cells in the presence of IL-2 and IL-15. (a, b) $CD3^+$ T cells were treated with epigenetic chemical probes as described in Supplementary Fig. 1 and further incubated for 4 days in the presence of IL-2, IL-15 and the individual chemical probes. Representative flow cytometry plots (a) and the mean fluorescence intensity (b) of CFSE in the $CD4^+$ and $CD8^+$ T cell populations on day 4 are shown (n=3, ordinary one-way ANOVA with Tukey's multiple comparisons test against the DMSO control). * P<0.05, ** P<0.01. Horizontal lines indicate the means ± s.d.

Supplementary Figure 4

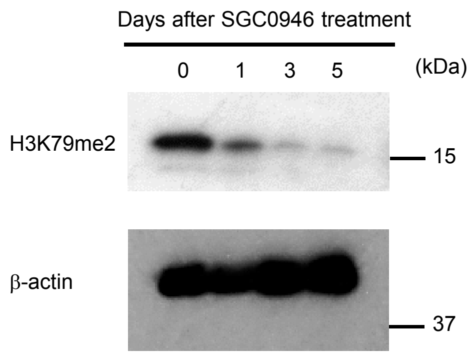

Supplementary Figure 4. Analysis of H3K79me2 in T cells treated with SGC0946. $CD3^+$ T cells were stimulated with aAPC/mOKT3 and treated with 0.5 μM SGC0946 on the next day. The global levels of H3K79me2 at the indicated time points were evaluated by immunoblotting. Representative blots of three experiments are shown.

Supplementary Figure 5

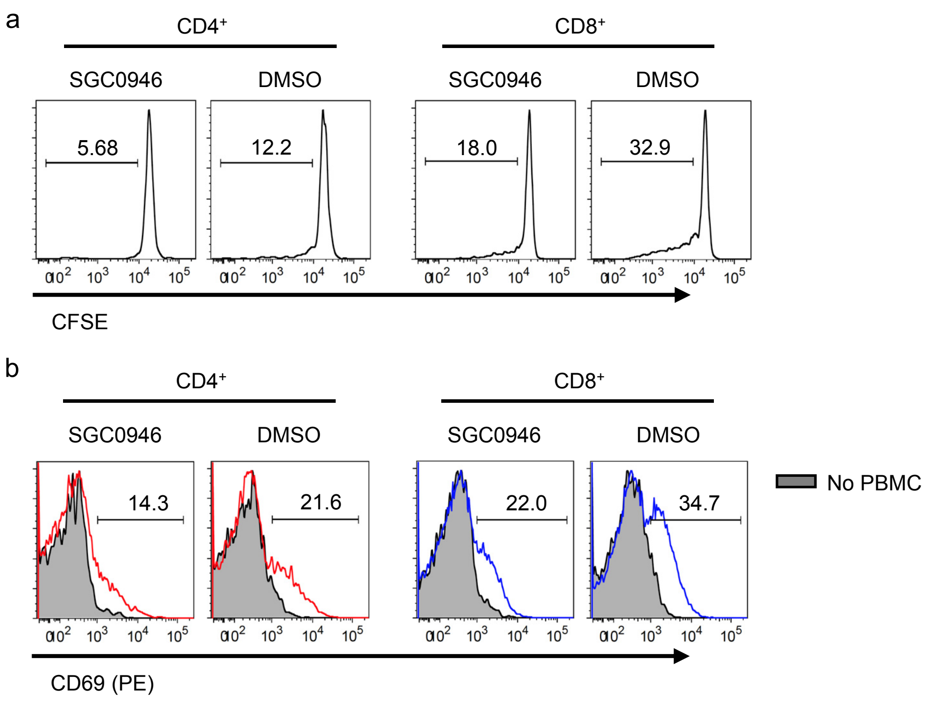

Supplementary Figure 5. Allogeneic T cell reactions are suppressed upon DOT1L inhibition. (a, b) $CD3^+$ T cells were cultured in the presence of 0.5 μM SGC0946 or DMSO and cocultured with allogeneic PBMC following CFSE labeling. Representative FACS plots analyzing CFSE dilution (a) and CD69 expression (b) four days after the coculture are shown (n=4 cultures).

Supplementary Figure 6

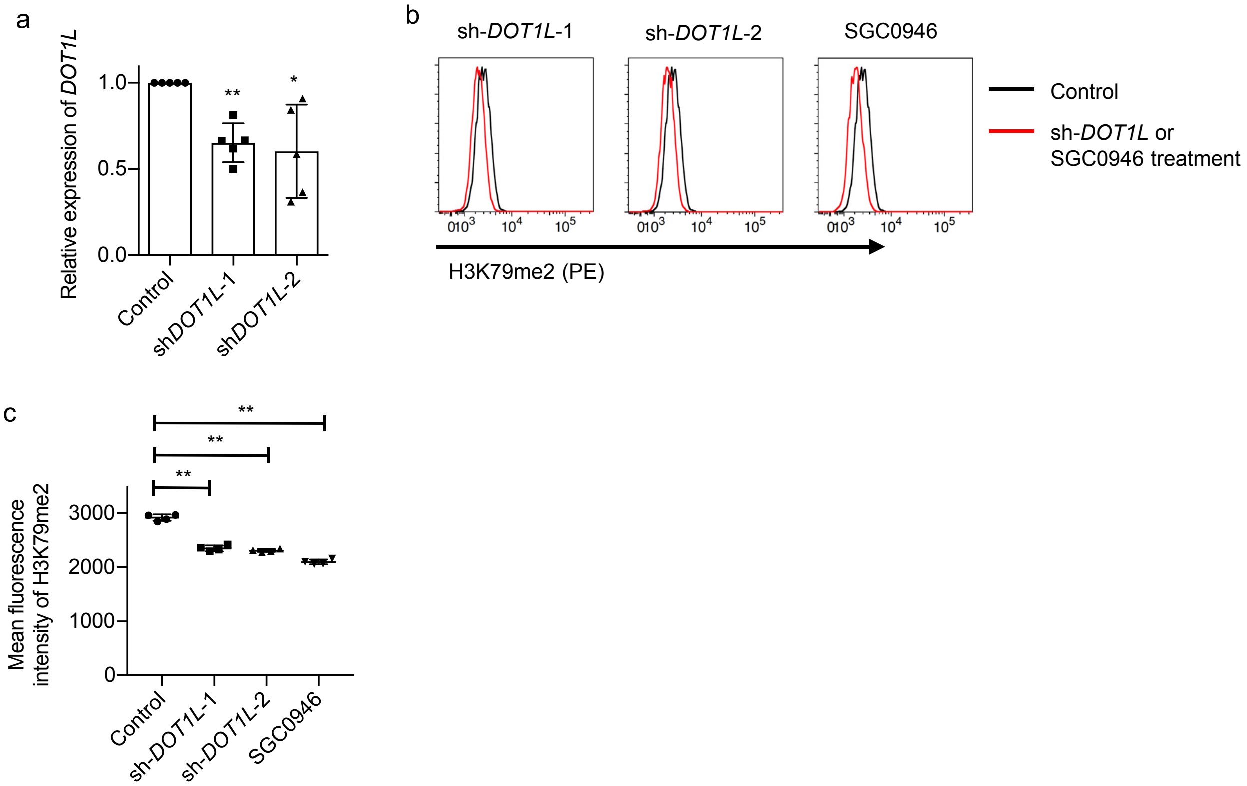

Supplementary Figure 6. Effects of DOT1L knockdown on H3K79me2 levels. (a-c) $CD3^+$ T cells were stimulated with aAPC/mOKT3 and were transduced with lentiviral shRNA against *DOT1L* or a control plasmid coexpressing ZsGreen. The control plasmid-transduced T cells were cultured in the presence or absence of SGC0946. $CD3^+$ $ZsGreen^+$ T cells were isolated, and *DOT1L* expression relative to the control was evaluated with quantitative PCR (a, n=5 samples; one-sample *t*-test compared to the control). (b, c) The global levels of H3K79me2 within the $ZsGreen^+$ T cell population were assessed with intracellular flow cytometry. Representative FACS plots (b) and the mean fluorescence intensity (c) are shown (n=4 cultures, ordinary one-way ANOVA with Tukey's multiple comparisons test). * $P<0.05$, ** $P<0.01$. Horizontal lines indicate the means ± s.d.

Supplementary Figure 7

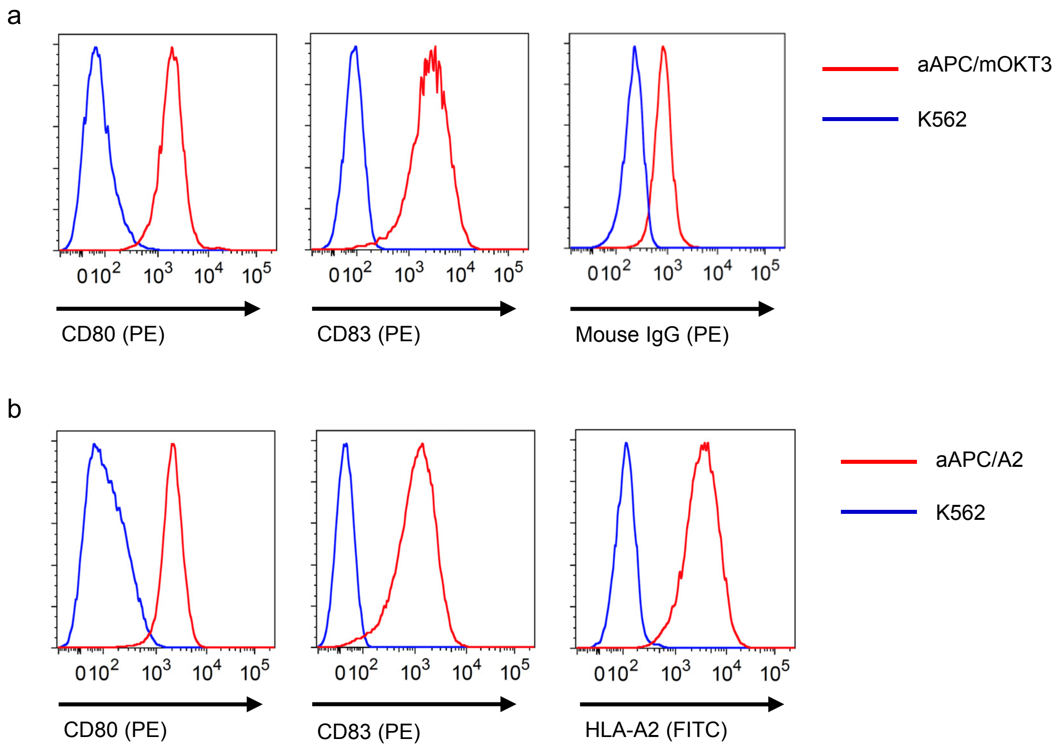

Supplementary Figure 7. K562-based artificial antigen presenting cells (aAPCs). (a, b) Surface expression of the indicated molecules in aAPC/mOKT3 (a) and aAPC/A2 (b). Expression of a membranous form of anti-CD3 mAb (clone OKT3) was detected using anti-mouse IgG.

Supplementary Figure 8

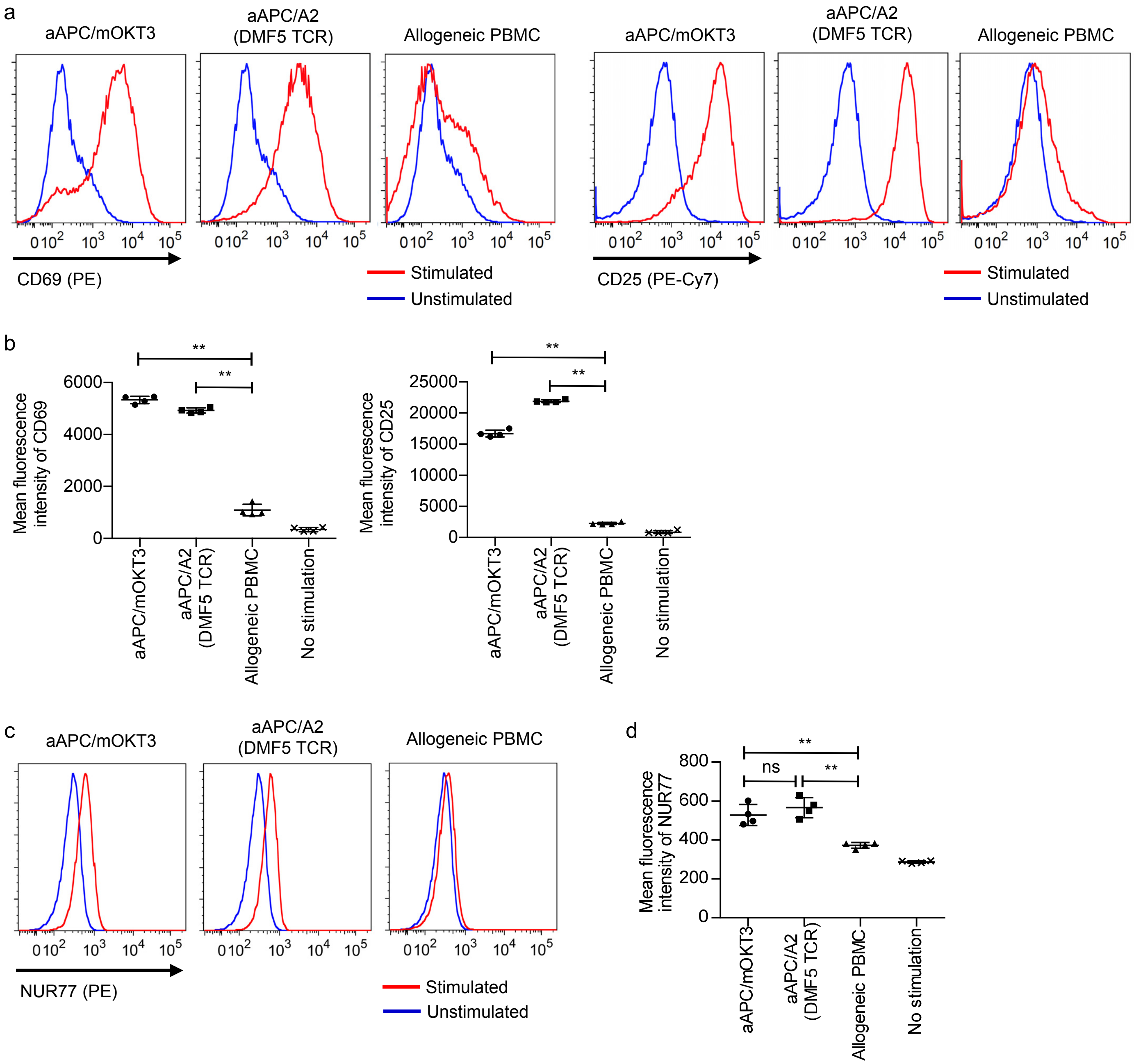

Supplementary Figure 8. Comparison of the strength of T cell activation by aAPCs and allogeneic PBMCs. (a-d) CD3$^+$ T cells were retrovirally transduced with control ΔNGFR or DMF5 TCR-P2A-ΔNGFR and cultured with the indicated cells. Surface expression of CD69 and CD25 (a), or intracellular expression of NUR77 (c) within the CD8$^+$ ΔNGFR$^+$ T cell population was analyzed on the next day. DMF5-transduced T cells were used for simulation by the aAPC/A2 loaded with MART1$_{27\text{-}35}$ peptide, and control ΔNGFR-transduced T cells for the other stimulations. The mean fluorescence intensity of each molecule is shown in (b) and (d) (n=4 technical replicates, ordinary one-way ANOVA with Tukey's multiple comparisons test). Horizontal lines indicate the means ± s.d. Similar results were reproduced in a repeated experiment. ** $P<0.01$. ns, not significant.

Supplementary Figure 9

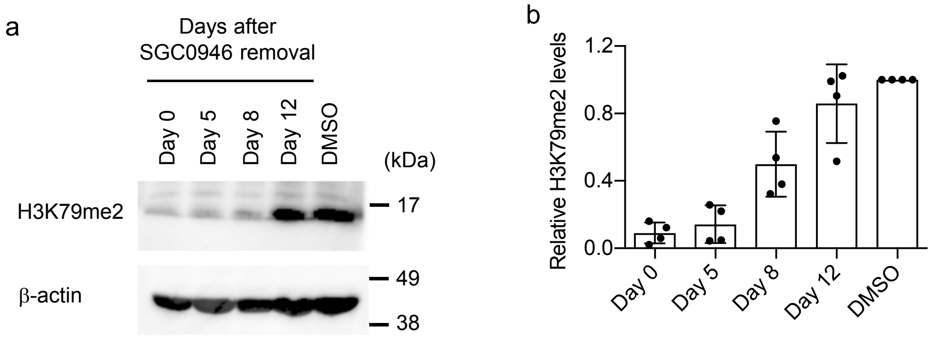

Supplementary Figure 9. Time-course assessment of H3K79me2 levels in SGC0946-treated T cells after withdrawal of the drug. (a, b) $CD3^+$ T cells were cultured in the presence of 0.5 μM SGC0946 for 7 days. On day 7, cells were re-plated in fresh medium and further cultured without the drug. The global levels of H3K79me2 were analyzed on the indicated days by immunoblotting (a). DMSO-treated control T cells were analyzed as the control. Relative H3K79me2 levels relative to β-actin at each time point compared with those in the control T cells are shown (b, n=4). Horizontal lines indicate the means ± s.d.

Supplementary Figure 10

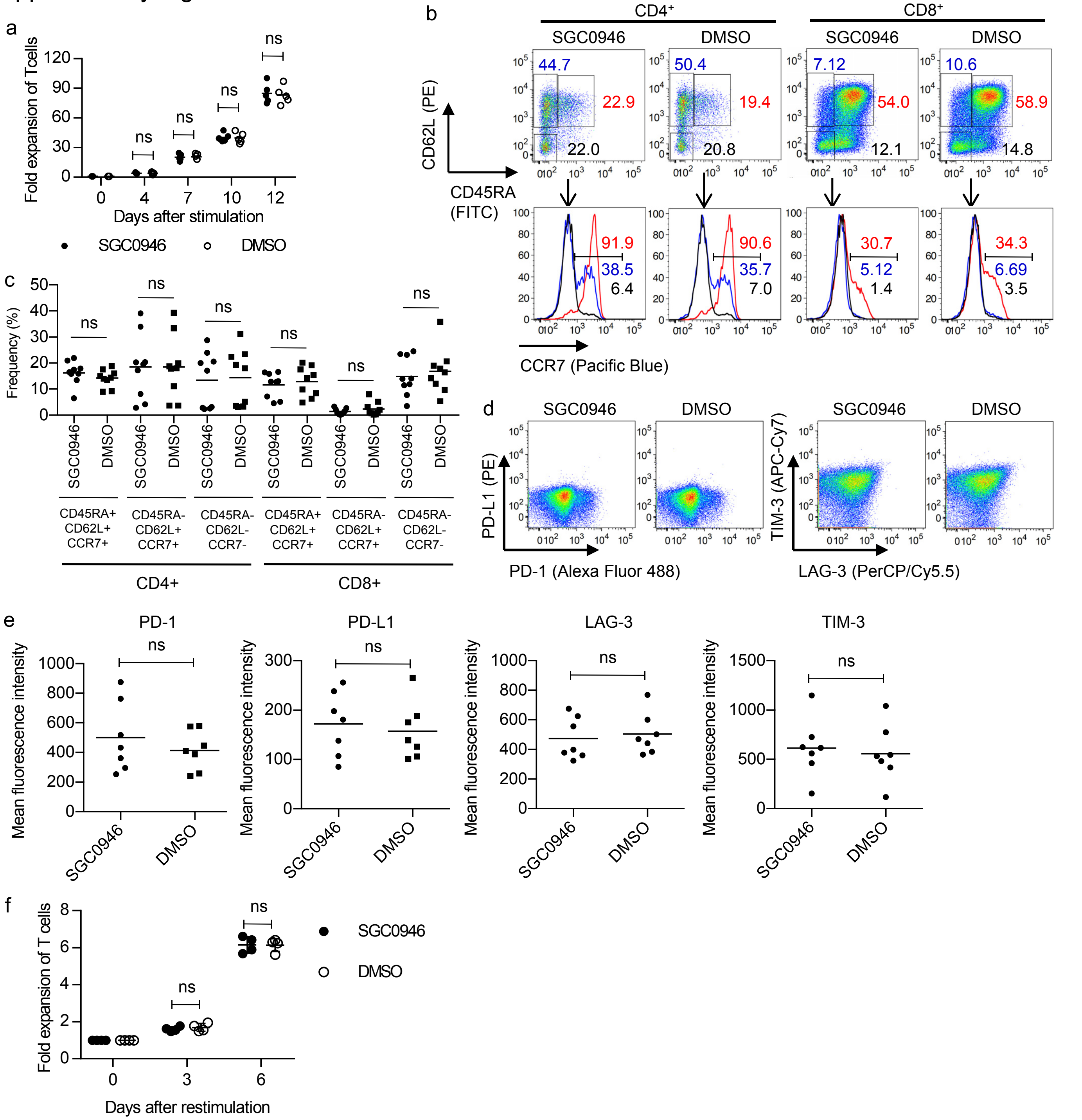

Supplementary Figure 10. Effects of SGC0946 treatment on T cell proliferation and differentiation. (a) CD3$^+$ T cells were stimulated with aAPC/mOKT3 (day 0). On the next day, T cells were split into two wells and cultured in the presence of IL-2 (100 IU per ml), IL-15 (10 ng per ml), and SGC0946 or DMSO. The fold expansion of T cells at each time point was calculated (n=5 different donor samples, paired two-sided *t*-test). (b, c) Surface expression of CD45RA, CD62L and CCR7 was analyzed on day 12. Representative FACS plots (b) and the frequencies of CD45RA$^+$ CD62L$^+$ CCR7$^+$, CD45RA$^-$ CD62L$^+$ CCR7$^+$, and CD45RA$^-$ CD62L$^-$ CCR7$^-$ cells within the CD4$^+$ or CD8$^+$ T cell population (c) are shown (n=9 different donor samples, paired two-sided *t*-test for each population). (d, e) Surface expression of T cell exhaustion markers (PD-1, PD-L1, LAG-3, and TIM-3) was analyzed on day 12. The data shown are representative FACS plots (d) and the mean fluorescence intensity of each marker in CD8$^+$ T cell population (e, n=7 different donor samples, paired two-sided *t*-test). (f) SGC0946- or DMSO-treated T cells were restimulated by aAPC/mOKT3 on day 12. The fold expansion of the restimulated T cells were calculated at the indicated time points (n=4 technical replicates, unpaired two-sided *t*-test). Similar results were reproduced with a different donor sample. Horizontal lines indicate the means ± s,d. ns, not significant.

Supplementary Figure 11

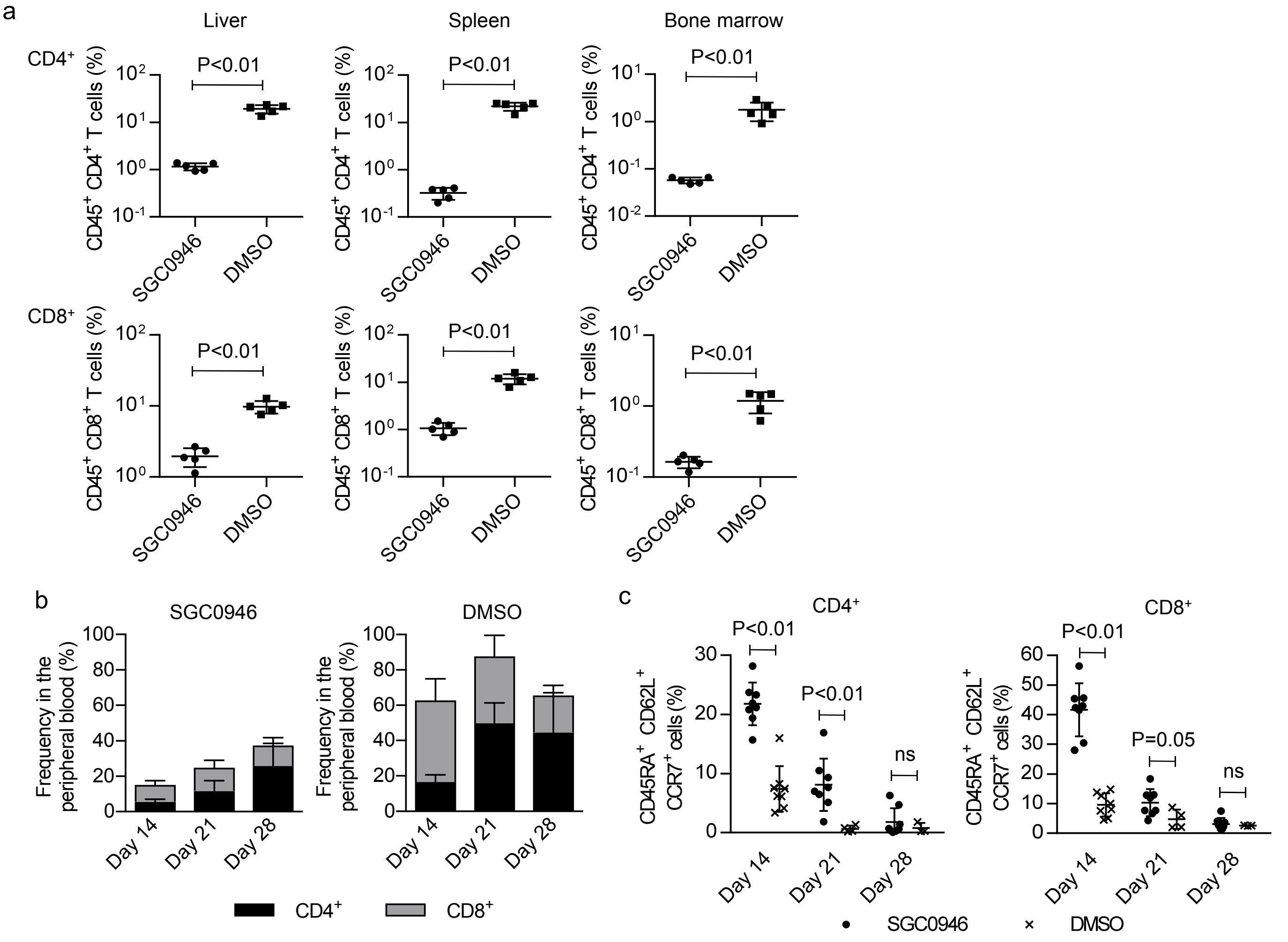

Supplementary Figure 11. Effects of SGC0946 treatment on the incidence of xenogeneic GVHD. (a) $CD3^+$ T cells were stimulated with aAPC/mOKT3, expanded in the presence of SGC0946 or DMSO and transplanted into irradiated NSG mice. Mice were euthanized on day 14, and the frequencies of $CD45^+$ $CD4^+$/$CD8^+$ human T cells in the liver, spleen and bone marrow were analyzed (n=5 mice, unpaired two-sided *t*-test). (b, c) Frequencies of $CD4^+$ and $CD8^+$ T cells in peripheral blood (b) and $CD45RA^+$ $CD62L^+$ $CCR7^+$ cells within human $CD4^+$ and $CD8^+$ T cell populations (c) in peripheral blood of mice transplanted with SGC0946- or DMSO-treated T cells at the indicated time points (n=8 mice, unpaired two-sided *t*-test). Horizontal lines indicate the means ± s,d. ns, not significant.

Supplementary Figure 12

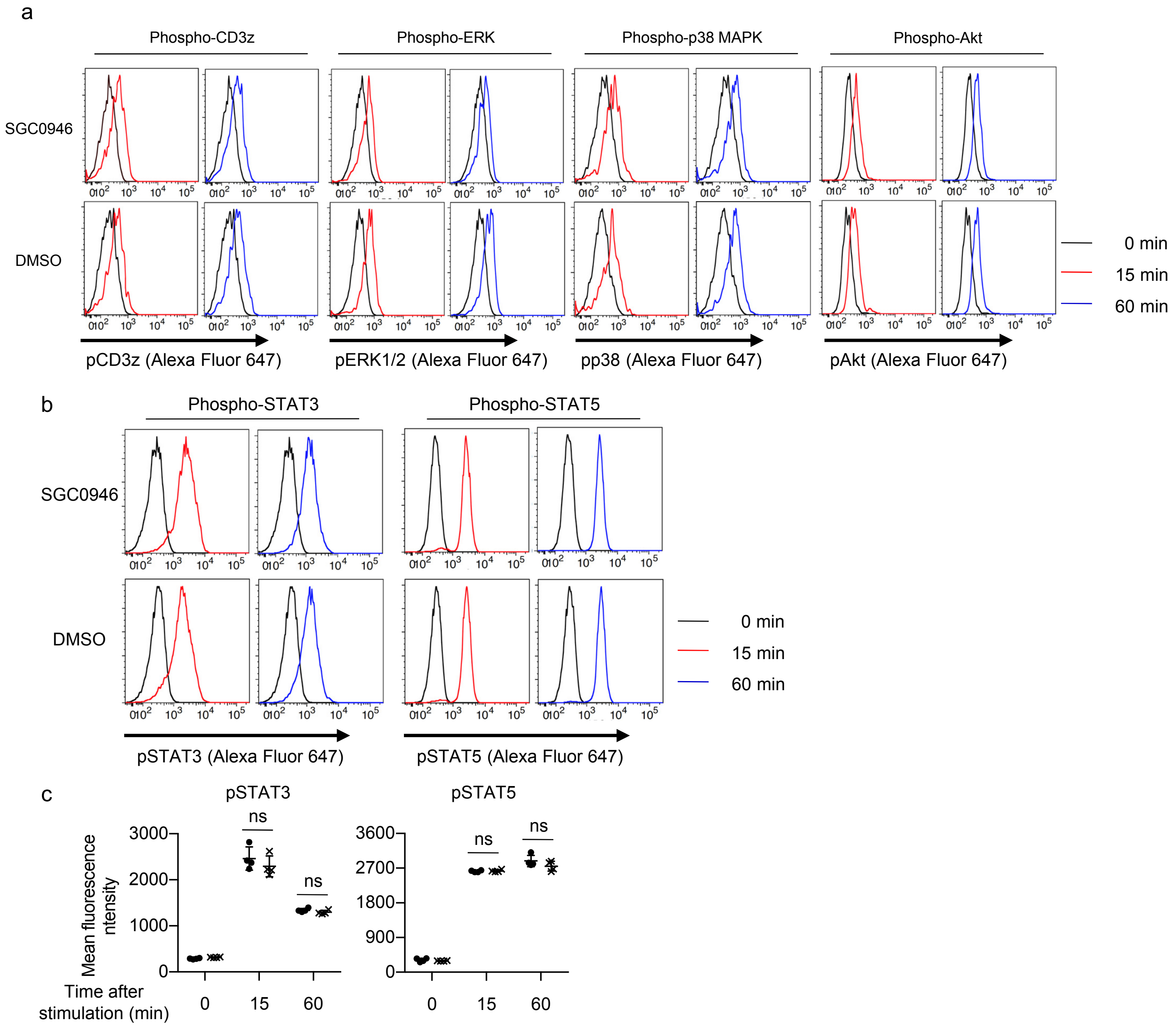

Supplementary Figure 12. Exploration of the signaling pathways affected by SGC0946 treatment. (a) $CD8^+$ T cells were retrovirally transduced with a low-affinity A2/MART1 T cell receptor (clone 413) and ΔNGFR, and cultured in the presence or absence of SGC0946 for 14 days. Cultured T cells were allowed to rest overnight in cytokine-free medium and then stimulated with aAPC/A2 loaded with 10 μg per ml A2/MART1$_{27\text{-}35}$ peptide. Phosphorylated CD3z, ERK, p38 MAPK, and Akt were analyzed by intracellular flow cytometry at the indicated time points. Representative FACS plots for four experiments are shown. (b, c) $CD8^+$ T cells were cultured with or without SGC0946 for two weeks and treated with 50 ng per ml IL-21 or 500 IU per ml IL-2 after resting overnight in cytokine-free medium. Phospho-STAT3 and STAT5 were analyzed by intracellular flow cytometry after IL-21 or IL-2 treatment, respectively. The data shown are representative FACS plots (b) and the mean fluorescence intensity (c, n=4, unpaired two-sided *t*-test for each time point). Horizontal lines indicate the means ± s.d. ns, not significant.

Supplementary Figure 13

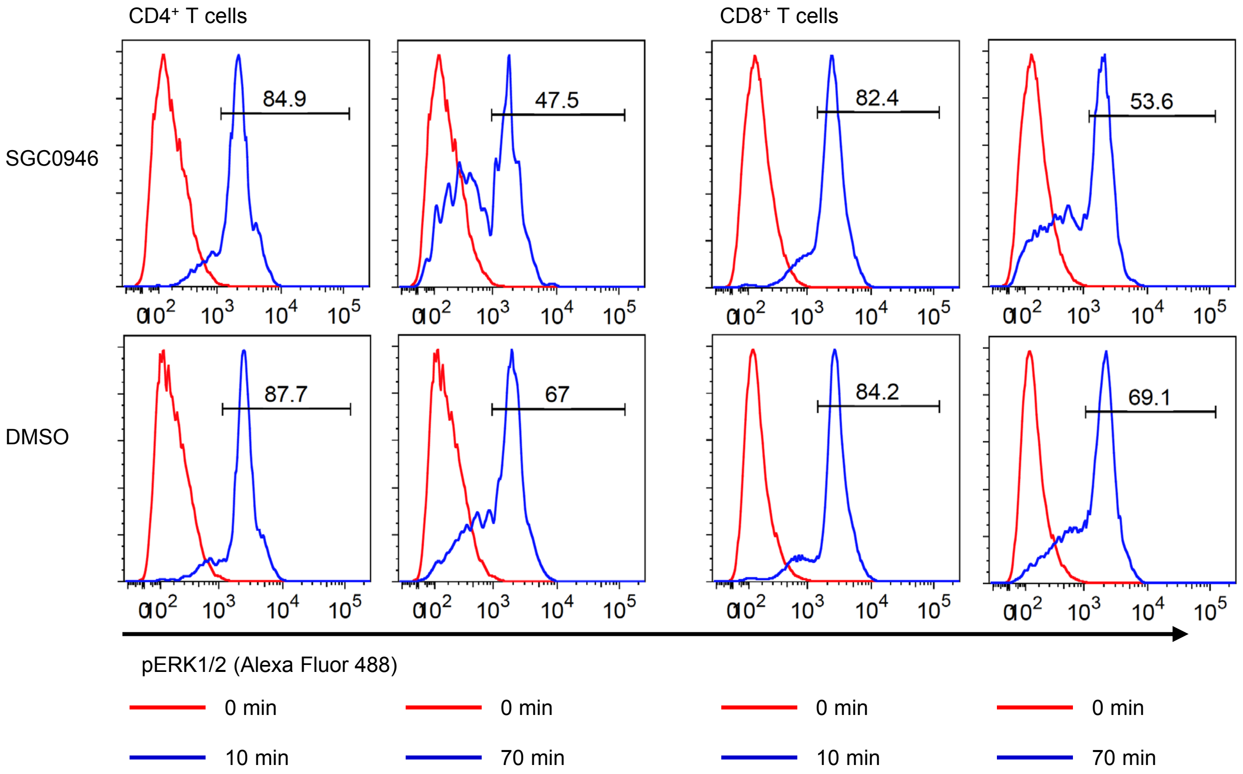

Supplementary Figure 13. Kinetics of ERK phosphorylation in stimulated T cells. $CD3^+$ T cells treated with SGC0946 or DMSO were incubated overnight in cytokine-free medium and then stimulated with PMA and ionomycin. Phosphorylation of ERK within the $CD4^+$ and $CD8^+$ T cell populations was analyzed with intracellular flow cytometry at the indicated time points. Representative FACS plots of four experiments are shown.

Supplementary Figure 14

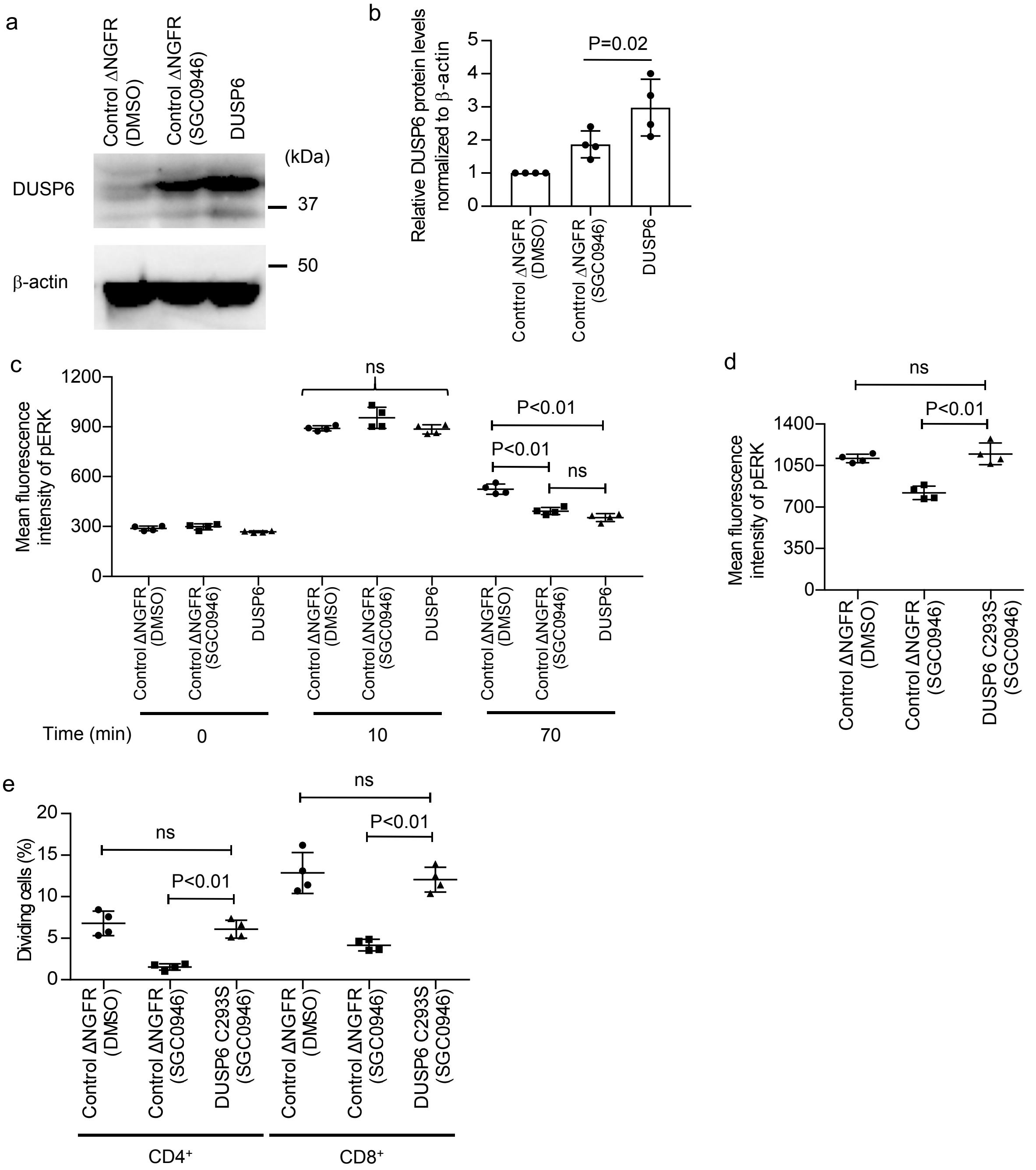

Supplementary Figure 14. DUSP6 promotes ERK dephosphorylation in stimulated T cells. (a) CD3$^+$ T cells were retrovirally transduced with DUSP6-IRES-ΔNGFR or control ΔNGFR. Control ΔNGFR-transduced cells were treated with DMSO or SGC0946. ΔNGFR$^+$ cells were isolated and subjected to immunoblotting analysis with an anti-DUSP6 antibody. Representative blots of four experiments (a) and the quantified DUSP6 protein levels normalized to β-actin are shown (b, n=4, paired two-sided *t*-test). (c) ERK phosphorylation after stimulation with PMA/ionomycin was evaluated in T cells transduced with control ΔNGFR or DUSP6. Control ΔNGFR-transduced cells were treated with DMSO or SGC0946. The mean fluorescence intensity (MFI) in the CD8$^+$ ΔNGFR$^+$ T cell population is shown (n=4 cultures, ordinary one-way ANOVA with Tukey's multiple comparisons test for each time point). (d,e) CD3$^+$ T cells were retrovirally transduced with a dominant–negative mutant of DUSP6 (C293S)-IRES-ΔNGFR or control ΔNGFR and treated with SGC0946 or DMSO. (d) ERK phosphorylation was analyzed 70 minutes after stimulation with PMA/ionomycin, and MFI in the CD8$^+$ ΔNGFR$^+$ T cell population was calculated (n=4 cultures, ordinary one-way ANOVA with Tukey's multiple comparisons test). (e) The indicated T cells were cocultured with allogeneic PBMC after being labeled with CFSE. The frequency of dividing cells in the ΔNGFR$^+$ T cell population was analyzed 4 days later (n=4 cultures, ordinary one-way ANOVA with Tukey's multiple comparisons test for each T cell population). Horizontal lines denote the means ± s.d. ns, not significant.

Supplementary Figure 15

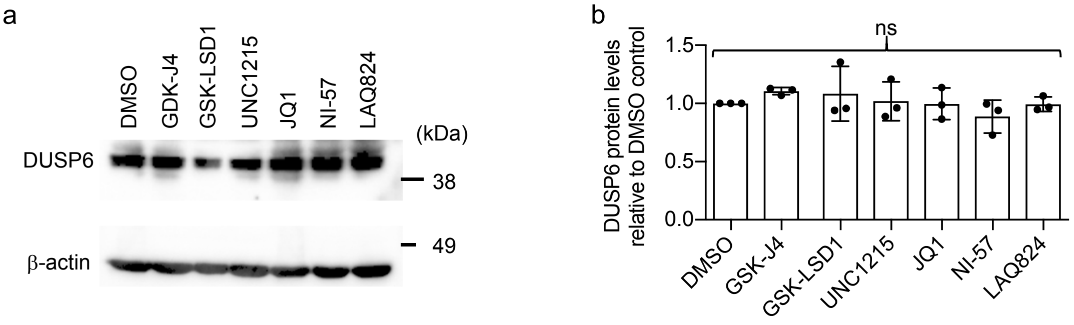

Supplementary Figure 15. Effects of different epigenetic chemical probes on DUSP6 expression levels. (a) $CD3^+$ T cells were treated with the indicated chemical probes for 14 days. The DUSP6 protein levels were analyzed by immunoblotting. Representative blots of three experiments (a) and the quantified DUSP6 protein levels normalized to β-actin are shown (b, n=3, repeated measures one-way ANOVA with Tukey's multiple comparisons test). Horizontal lines indicate the means ± s.d. ns, not significant.

Supplementary Figure 16

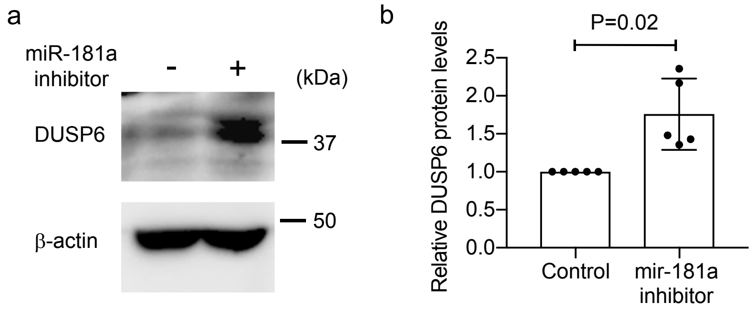

Supplementary Figure 16. DUSP6 expression upon inhibition of miR-181a. (a, b) $CD3^+$ T cells were transfected with control or a miR-181a inhibitor. DUSP6 expression was analyzed by immunoblotting 48 hours after transfection. Representative blots of five experiments (a) and the quantified DUSP6 protein levels normalized to β-actin are shown (b, n=5, one sample *t*-test). Horizontal lines indicate the means ± s.d.

Supplementary Figure 17

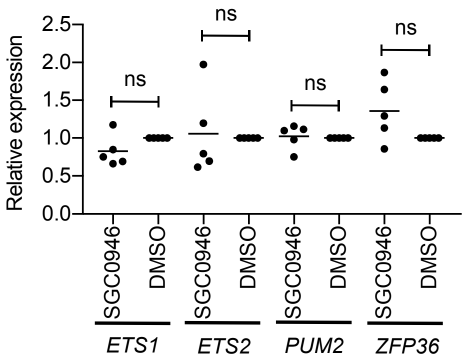

Supplementary Figure 17. Expression analysis of genes that regulate DUSP6 expression. $CD3^+$ T cells were treated with SGC0946 or DMSO for 14 days. The expression levels of the indicated genes in the SGC0946-treated T cells relative to the DMSO-treated control T cells were analyzed by qPCR (n=5 different samples, one-sample *t*-test). Horizontal lines indicate the mean values. ns, not significant.

Supplementary Figure 18

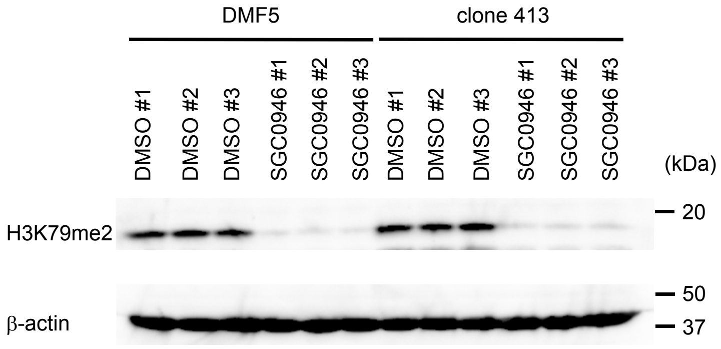

Supplementary Figure 18. Analysis of the H3K79me2 levels in DMSO- or SGC0946-treated CD8$^+$ T cells. CD8$^+$ T cells derived from three healthy donors were transduced with the DMF5 or clone 413 A2/MART1 TCR and treated with SGC0946 or DMSO (control) for 10 days. Repression of the H3K79me2 levels in SGC0946-treated T cells was confirmed by immunoblotting.

Supplementary Figure 19

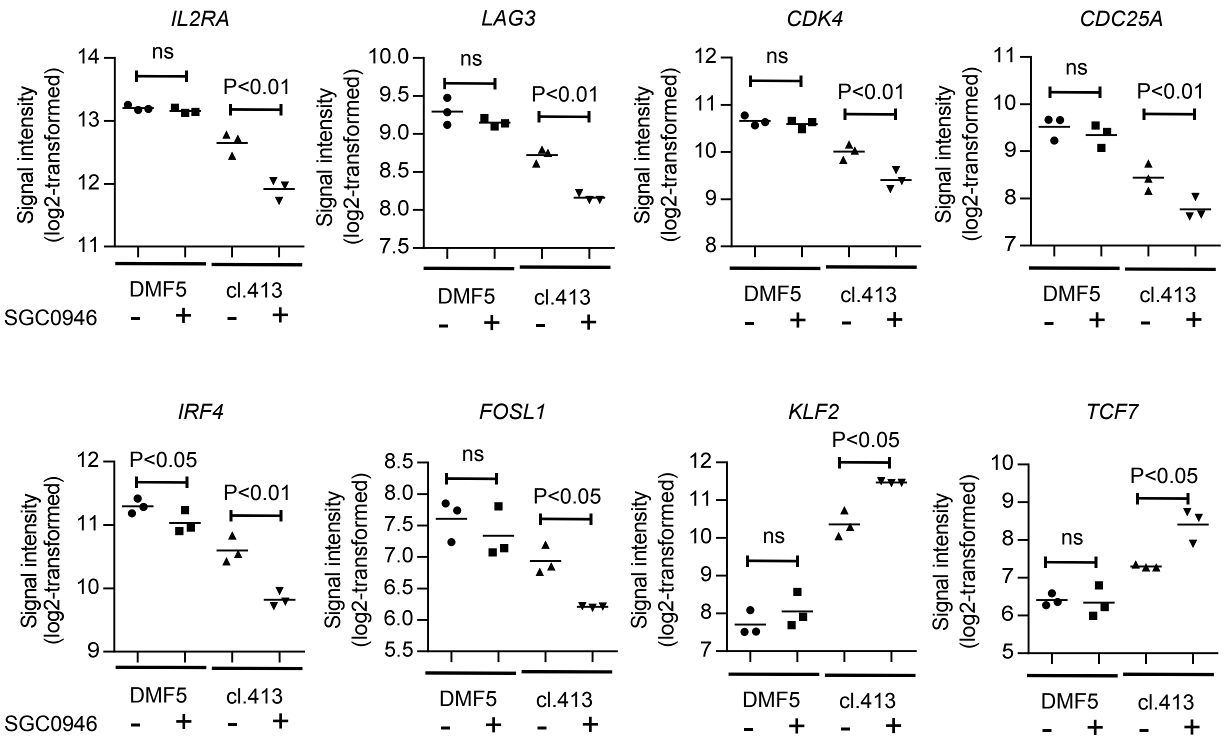

Supplementary Figure 19. Gene expression profiles of the SGC0946-treated T cells after high- or low-avidity T cell stimulation. DMF5 or clone 413 A2/MART1 TCR-transduced $CD8^+$ T cells were treated with SGC0946 or DMSO (control) and stimulated with aAPC/A2 loaded with heteroclitic A2/$MART1_{27\text{-}35}$ peptide. Gene expression profiles of stimulated T cells were compared by microarray analysis. The expression levels of the representative genes that are differentially expressed between the SGC0946-treated and control clone 413 TCR-transduced T cells (n=3 different donor samples, paired two-sided *t*-test). Horizontal lines indicate the mean values. ns, not significant.

Supplementary Figure 20

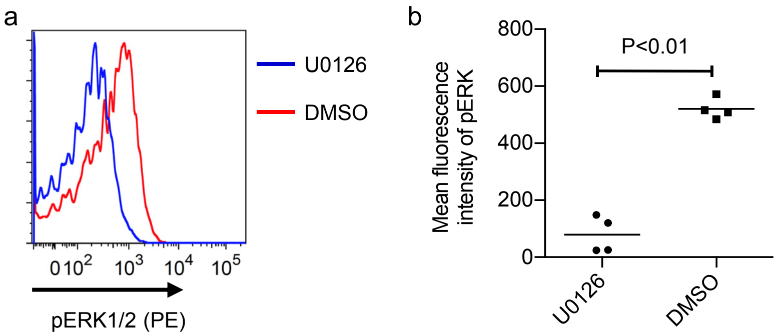

Supplementary Figure 20. MEK inhibition efficiently inhibits ERK phosphorylation in high-avidity T cells. (a, b) DMF5 A2/MART1 TCR-transduced $CD8^+$ T cells were stimulated with aAPC/A2 loaded with 10 µg per ml A2/$MART1_{27\text{-}35}$ peptide in the presence or absence of 2.5 µM U0126, and the pERK levels were analyzed 10 minutes following stimulation. Representative FACS plots (a) and the mean fluorescence intensity of pERK (b) are shown (n=4 different samples, paired two-sided *t*-test). Horizontal lines indicate the mean values.

Supplementary Figure 21

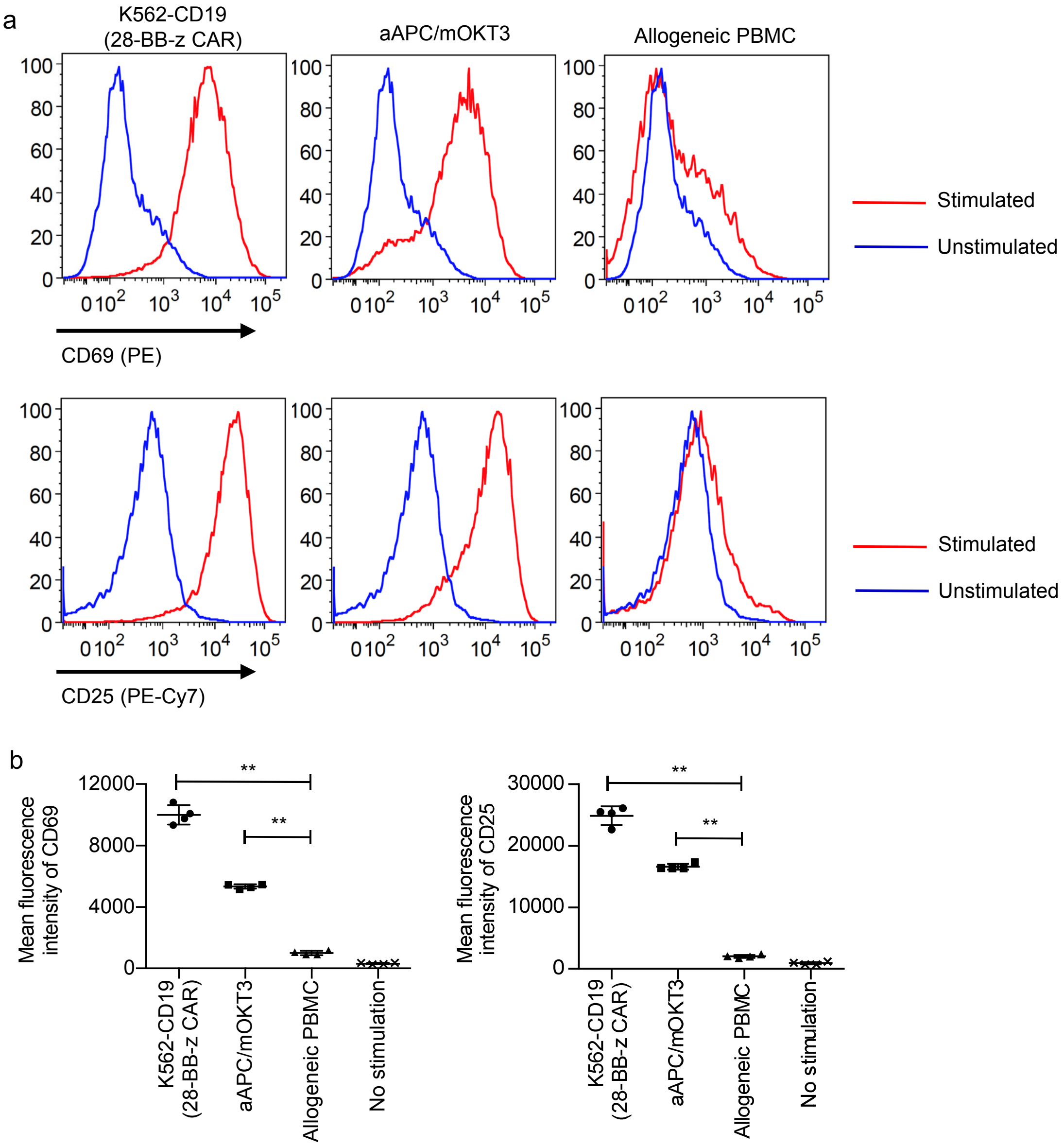

Supplementary Figure 21. T cell activation mediated by a chimeric antigen receptor (CAR). (a, b) $CD3^+$ T cells were retrovirally transduced with control ΔNGFR or ΔNGFR-P2A-anti-CD19 CAR with CD28 and 4-1BB domains (28-BB-z) and stimulated with the indicated cells. Surface expression of CD69 and CD25 within the $CD8^+$ $\Delta NGFR^+$ T cell population was analyzed the next day. CAR-transduced T cells were cultured with K562 expressing CD19 (K562-CD19), while control ΔNGFR-transduced T cells were stimulated with aAPC/mOKT3 or allogeneic PBMC. Representative FACS plots (a) and the mean fluorescence intensity for each expression are shown (b, n=4 technical replicates, ordinary one-way ANOVA with Tukey's multiple comparisons test). Horizontal lines indicate the means ± s.d. Similar results were reproduced in a repeated experiment. ** $P<0.01$.

Supplementary Figure 22

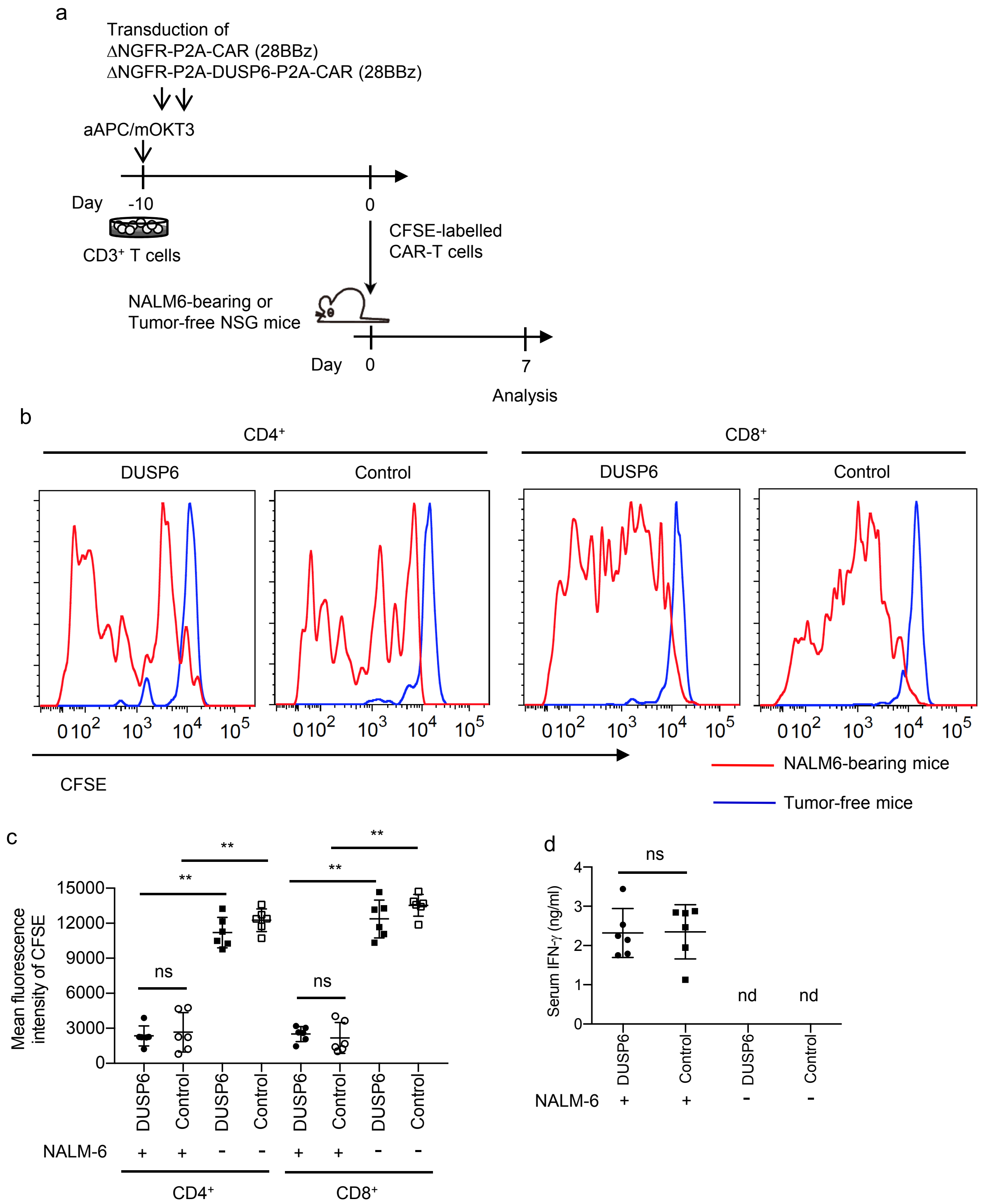

Supplementary Figure 22. DUSP6 overexpression in CAR-T cells do not impair CAR-T cell functions *in vivo*. (a) NALM6-bearing or tumor-free mice were treated with 6 million CFSE-labelled 28-BB-z CAR-T cells with or without overexpression of DUSP6 (day 0). Mice were sacrificed on day 7, and analyzed for CFSE dilution of CAR-T cells within the spleen. (b, c) Representative FACS plots analyzing the CFSE dilution (b) and mean fluorescence intensity of CFSE in the ΔNGFR$^+$ CD4$^+$/CD8$^+$ T cell population (c, n=6 mice for each condition, ordinary one-way ANOVA with Tukey's multiple comparisons test). (d) Serum was collected from NALM6-bearing or tumor-free mice at day 3 after CAR-T cell infusion. The serum concentration of IFN-γ was measured by ELISA (n=6 mice for each, unpaired two-sided *t*-test). ** P<0.01. ns, not significant; nd, not detected. In **c** and **d**, Horizontal lines denote the means ± s.d.

Supplementary Figure 23

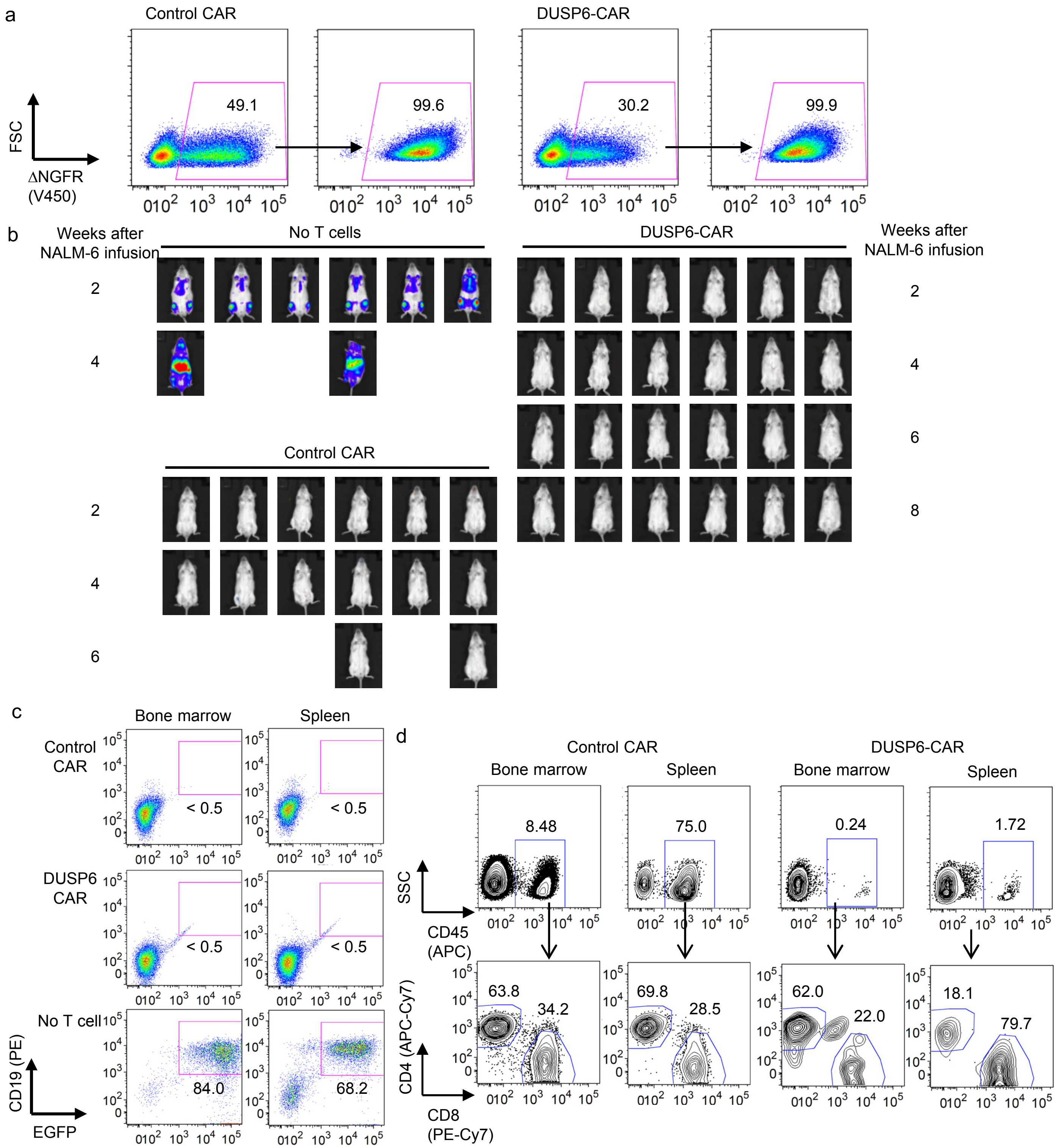

Supplementary Figure 23. Treatment of the CD19$^+$ acute lymphoblastic leukemia cell line NALM-6 with anti-CD19 CAR-engineered T cells with or without ectopic expression of DUSP6. (a) CD3$^+$ T cells were retrovirally transduced with an anti-CD19 CAR gene and ΔNGFR, or CAR, DUSP6 and ΔNGFR linked to a Furin-SGSG-P2A sequence. ΔNGFR$^+$ cells were magnetically isolated, and their purity was analyzed by flow cytometry. (b) NSG mice were intravenously infused with NALM-6 expressing EGFP-luciferase (NALM6-GL) and treated with control or DUSP6-coexpressing CAR-T cells 3 and 5 days following leukemia infusion (3 million CAR-T cells per infusion). *In vivo* bioluminescent imaging of the luciferase activity at the indicated time points after leukemia cell infusion is shown. (c, d) Autopsy analysis of untreated mice and those infused with CAR-T cells. The data shown are representative FACS plots evaluating the persistence of NALM6-GL (c) and CAR-T cells (d) in the bone marrow and spleen.

Supplementary Figure 24

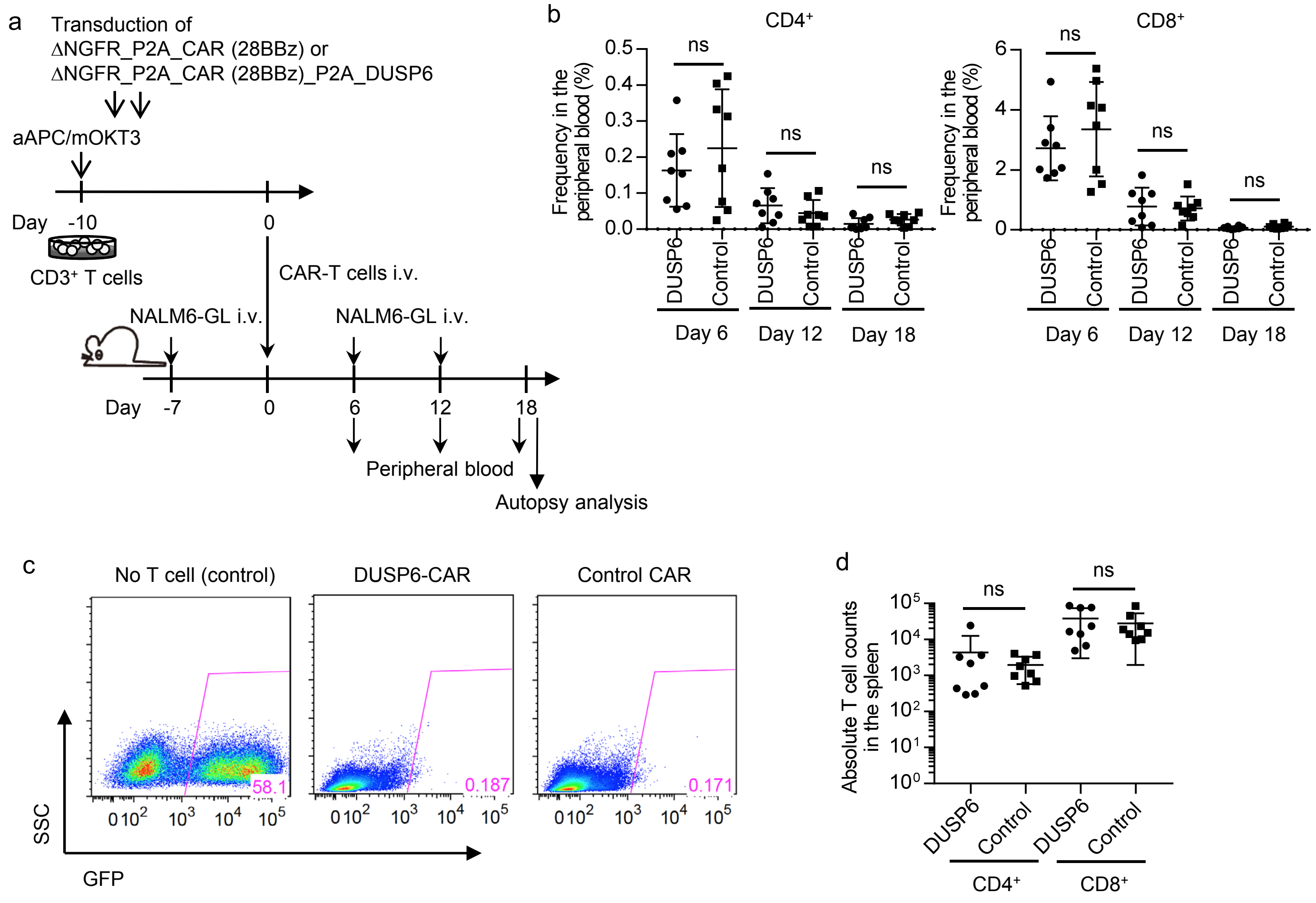

Supplementary Figure 24. DUSP6 overexpression in CAR-T cells does not compromise antitumor effects upon tumor rechallenge *in vivo*. (a) NALM6-bearing leukemia mice were treated with 6 million CAR-T cells with or without overexpression of DUSP6 (day 0). NALM6-GL was reinjected into the mice on days 6 and 12, and the persistence of CAR-T cells and leukemia progression were analyzed. (b) The persistence of CAR-T cells in the peripheral blood was analyzed on days 6, 12 and 18 (n=8 mice for each group, unpaired two-sided *t*-test). (c, d) On day 18, mice were sacrificed and analyzed for infiltration of NALM6-GL and the persistence of CAR-T cells in the spleen. The data shown are representative FACS plots analyzing the frequency of GFP^+ leukemia cells (c, n=4 mice for "No T cell control" group, n=8 mice each for CAR-T cell treatment group) and the absolute number of the persisting CAR-T cells in the spleen (d, n=8 mice, unpaired two-sided *t*-test). ns, not significant. Horizontal lines indicate the means ± s.d.

Supplementary Figure 25

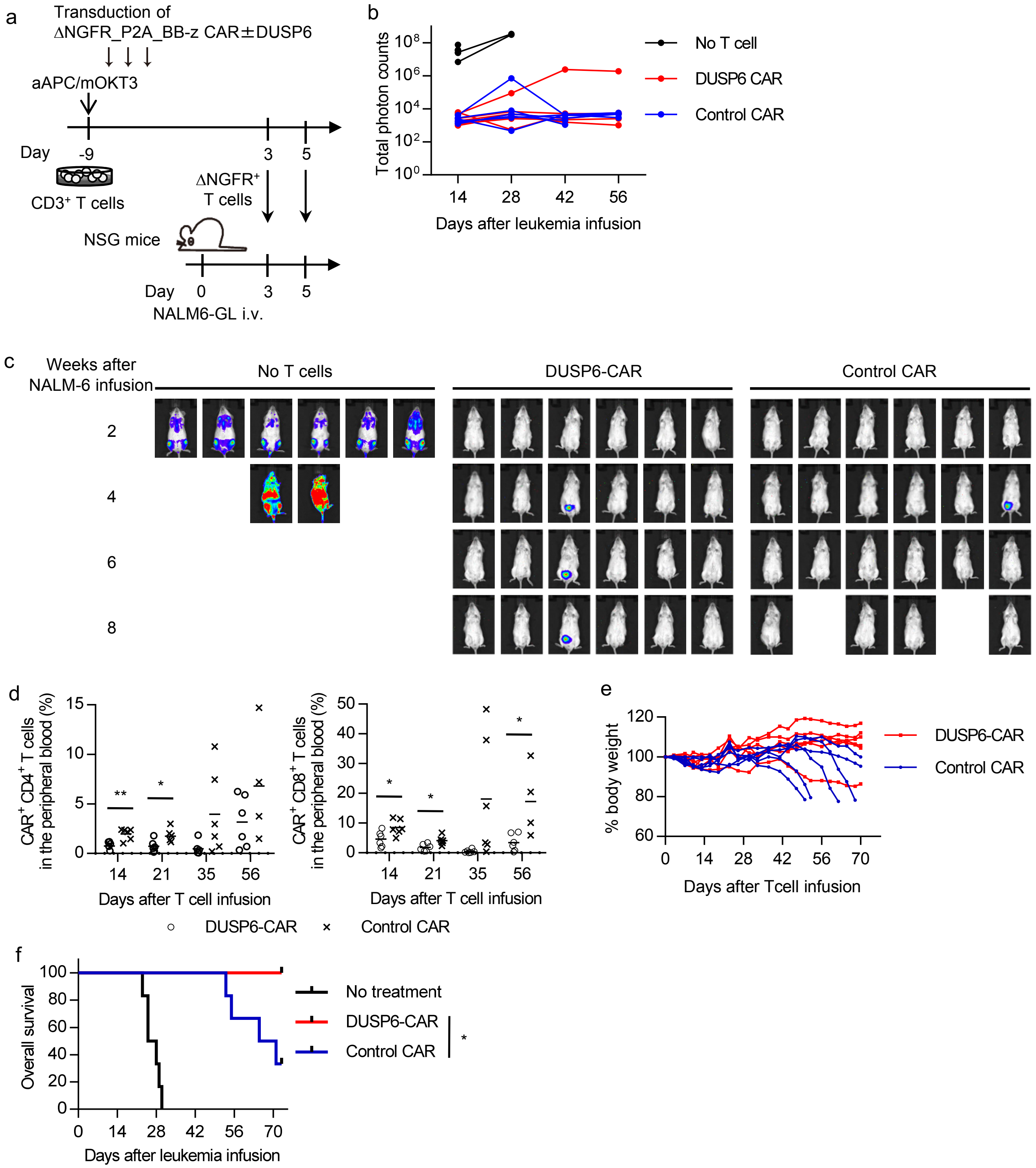

Supplementary Figure 25. Treatment of the CD19^{+} acute lymphoblastic leukemia cell line NALM-6 with anti-CD19 BB-z CAR-engineered T cells with or without ectopic expression of DUSP6. (a) CD3^{+} T cells were retrovirally transduced with an anti-CD19 CAR gene that had a 4-1BB signaling domain (BB-z) and ΔNGFR, or BB-z CAR, DUSP6 and ΔNGFR linked to a Furin-SGSG-P2A sequence. ΔNGFR^{+} cells were magnetically isolated and infused into NALM6-GL-bearing NSG mice using the same protocol described in Fig. 6a (3 million CAR-T cells per infusion). (b, c) Leukemia progression was monitored by *in vivo* bioluminescent imaging of the luciferase activity. The data shown are the total photon counts (b) and images of individual mice (c) at the indicated time points. (d) The frequencies of human CD45^{+} CD4^{+}/CD8^{+} CAR-T cells in the peripheral blood (n=6 mice, unpaired two-sided *t*-test). Horizontal lines indicate the mean values. (e) Sequential monitoring of body weight relative to the weight on day 0. (f) Kaplan-Meier analysis for overall survival after NALM6-GL infusion (n=6 mice, log-rank test). * P<0.05, ** P<0.01.

Supplementary Figure 26

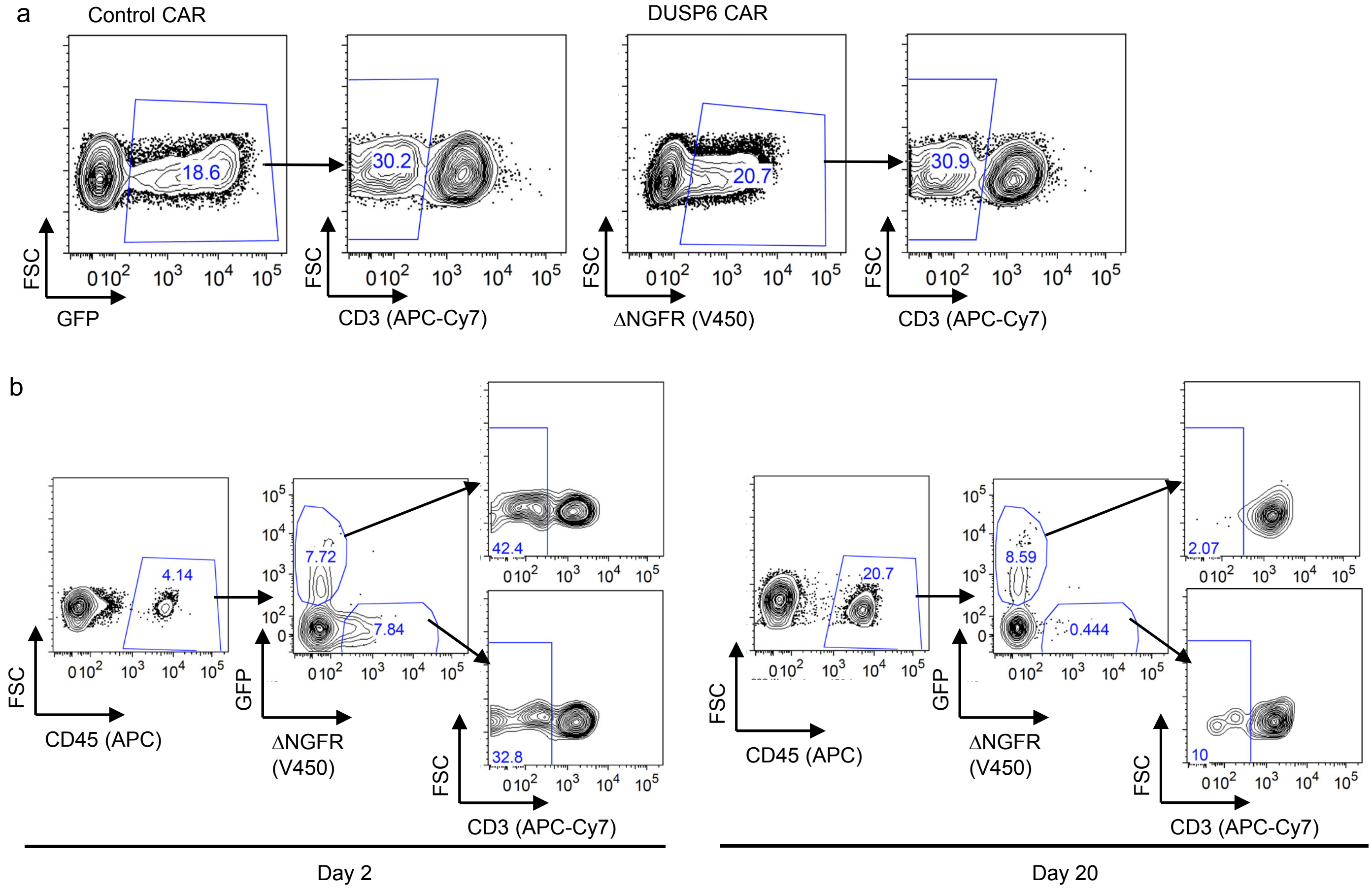

Supplementary Figure 26. DUSP6 overexpression suppresses endogenous TCR-mediated xenogeneic GVHD. (a) $CD3^+$ T cells were individually transduced with EGFP-P2A-BB-z CAR or ΔNGFR-P2A-BB-z CAR-P2A-DUSP6, and endogenous TCR expression was ablated by CRISPR/Cas9. NALM6-bearing leukemia mice were coinfused with $CD3^{+/-}$ control CAR-T cells ($EGFP^+$) and $CD3^{+/-}$ DUSP6-CAR-T cells ($ΔNGFR^+$). Representative FACS plots showing CD3 expression in control CAR ($EGFP^+$) or DUSP6-CAR ($ΔNGFR^+$) T cells before transplantation. (b) The persistence of CAR-T cells was monitored in the peripheral blood of treated mice. Representative FACS plots of 6 mice showing the frequency of each CAR-T cell population.

Supplementary Figure 27

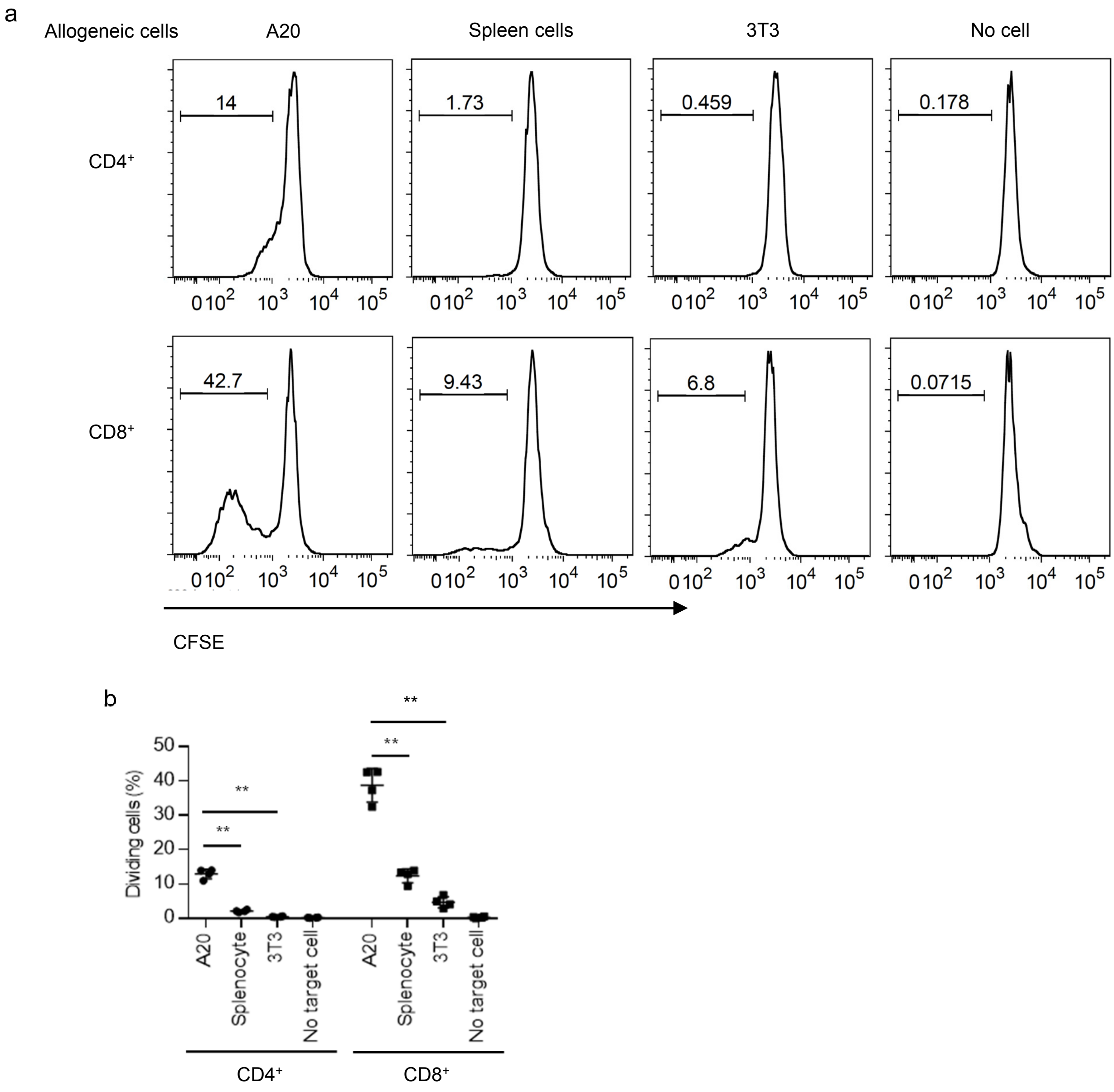

Supplementary Figure 27. Comparison of the allogeneic T cell responses between tumor cells and normal cells. (a) $CD3^+$ T cells derived from C57BL/6 mice were primed with anti-CD3/CD28 beads. On day 4, CFSE-labelled T cells were cultured with A20 lymphoma cells, Balb/c splenocytes or Balb/c-derived 3T3 cells, and the CFSE dilution was analyzed 4 days later. Representative FACS plots (a) and the frequency of CFSE-diluting cells are shown (b, n=4 cultures, ordinary one-way ANOVA with Tukey's multiple comparisons test). Horizontal lines denote the means ± s.d. ** P<0.01.

Supplementary Figure 28

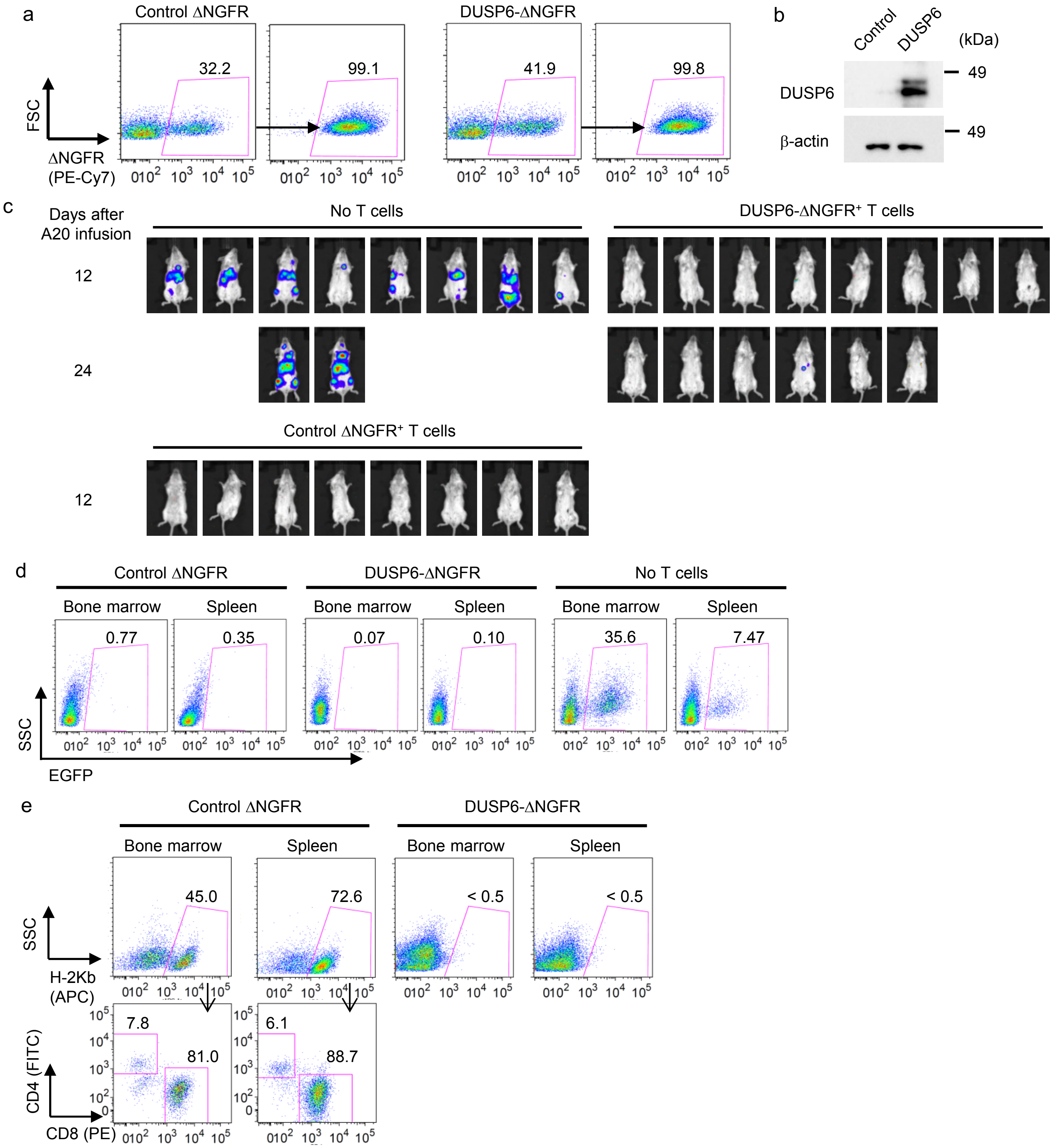

Supplementary Figure 28. Effects of DUSP6 overexpression on the development of graft-versus-tumor and graft-versus-host disease by allogeneic T cells in a mouse tumor model. (a, b) $CD3^+$ T cells derived from C57BL/6 mice were retrovirally transduced with ΔNGFR or codon-optimized *Dusp6* and ΔNGFR linked to a Furin-SGSG-P2A sequence. $\Delta NGFR^+$ cells were magnetically isolated and analyzed for purity by flow cytometry (a). Expression of DUSP6 in the isolated samples was evaluated by immunoblotting (b). (c) Sublethally irradiated Balb/c mice were transplanted with the A20 lymphoma cell line expressing the EGFP-luciferase fusion gene (A20-GL) and C57BL/6 mouse-derived $CD3^+$ T cells expressing ΔNGFR or DUSP6-ΔNGFR. *In vivo* bioluminescent imaging of the luciferase activity at the indicated time points is shown. (d, e) Autopsy analysis of the Balb/c mice untreated or treated with control or DUSP6-expressing T cells. Representative FACS plots evaluating the persistence of A20-GL (d) and transplanted T cells (e) in the bone marrow and spleen (n=8 mice for each group).

Supplementary Figure 29

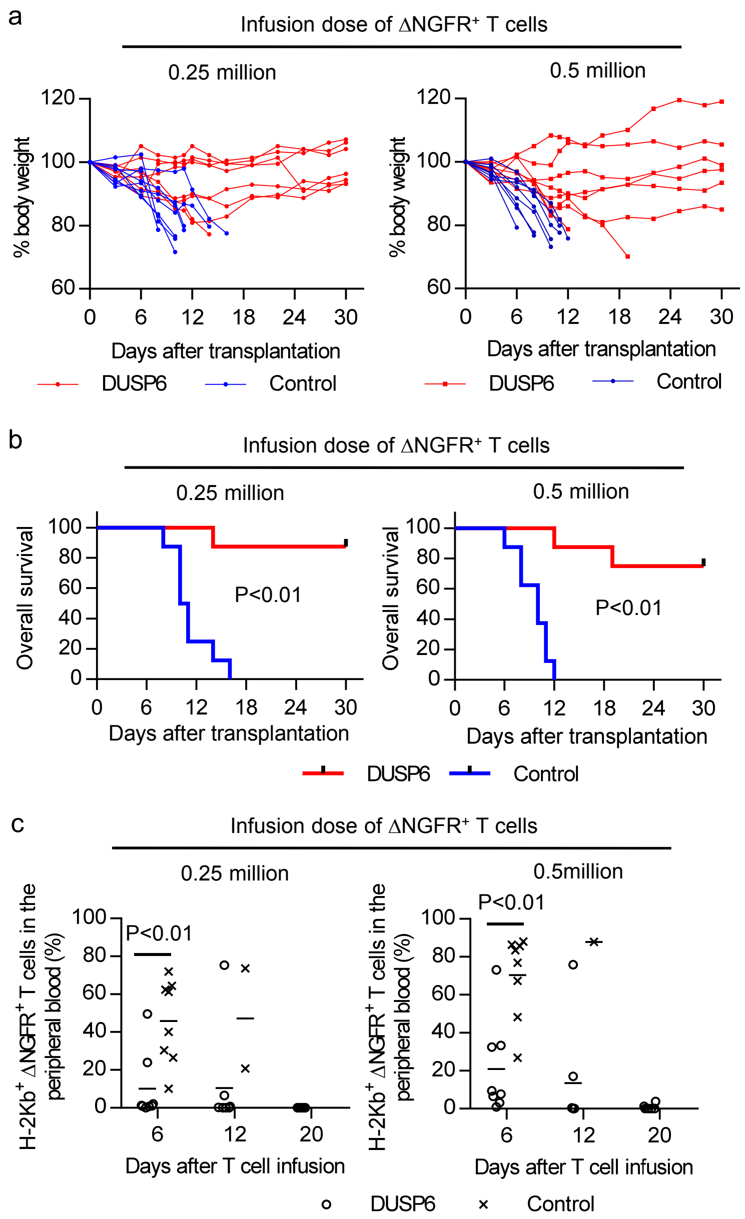

Supplementary Figure 29. Dose escalation of infused allogeneic T cells. (a-c) CD3+ T cells from C57BL/6 mice were retrovirally transduced with ΔNGFR, or codon-optimized *Dusp6* and ΔNGFR linked to a Furin-SGSG-P2A sequence. Isolated ΔNGFR+ T cells (2.5 x 10^5 or 5 x 10^5) together with A20-GL were infused into irradiated Balb/c mice. Serial monitoring of body weight (a), overall survival (b), and frequency of H-2Kb+ ΔNGFR+ T cells in transplanted mice are shown (n=8 mice for each group; b, log-rank test; c, unpaired two-sided *t*-test). The data shown are a composite of three independent experiments. Horizontal lines indicate the mean values.

Supplementary Figure 30

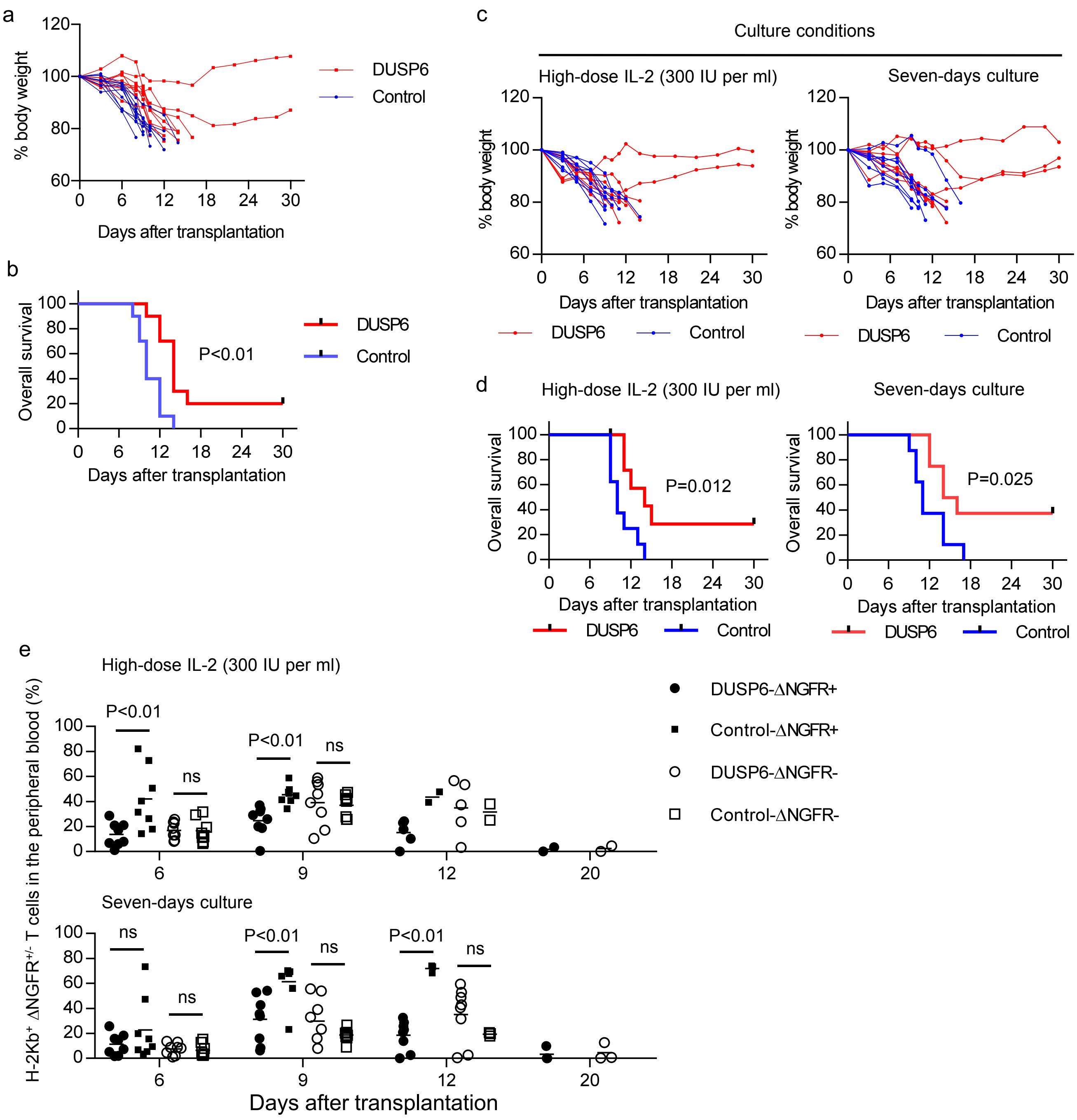

Supplementary Figure 30. Comparison of allogeneic T cell proliferation between DUSP6-transduced and untransduced T cells. (a, b) CD3^{+} T cells from C57BL/6 mice were retrovirally transduced with control ΔNGFR, or DUSP6-P2A-ΔNGFR and infused into irradiated Balb/c mice as described in Fig. 7f. Serial monitoring of body weight (a) and overall survival (b) of transplanted mice (n=10 mice for each group, log-rank test). (c, d) C57BL/6-derived CD3^{+} T cells were transduced with control ΔNGFR, or DUSP6-P2A-ΔNGFR and cultured with 300 IU per ml IL-2 for 4 days or with 20 IU per ml IL-2 and 10 ng per ml IL-15 for 7 days, and 3 x 10^{5} ΔNGFR$^{+/-}$ T cells were transplanted into irradiated Balb/c mice. Serial monitoring of body weight (c) and overall survival (d) of transplanted mice are shown (n=8 mice for each group; d, log-rank test). (e) The frequency of ΔNGFR$^{+/-}$ donor T cells cultured under the indicated conditions was monitored in peripheral blood (n=8, unpaired two-sided *t*-test). Horizontal lines indicate the mean values. ns, not significant.

Supplementary Figure 31

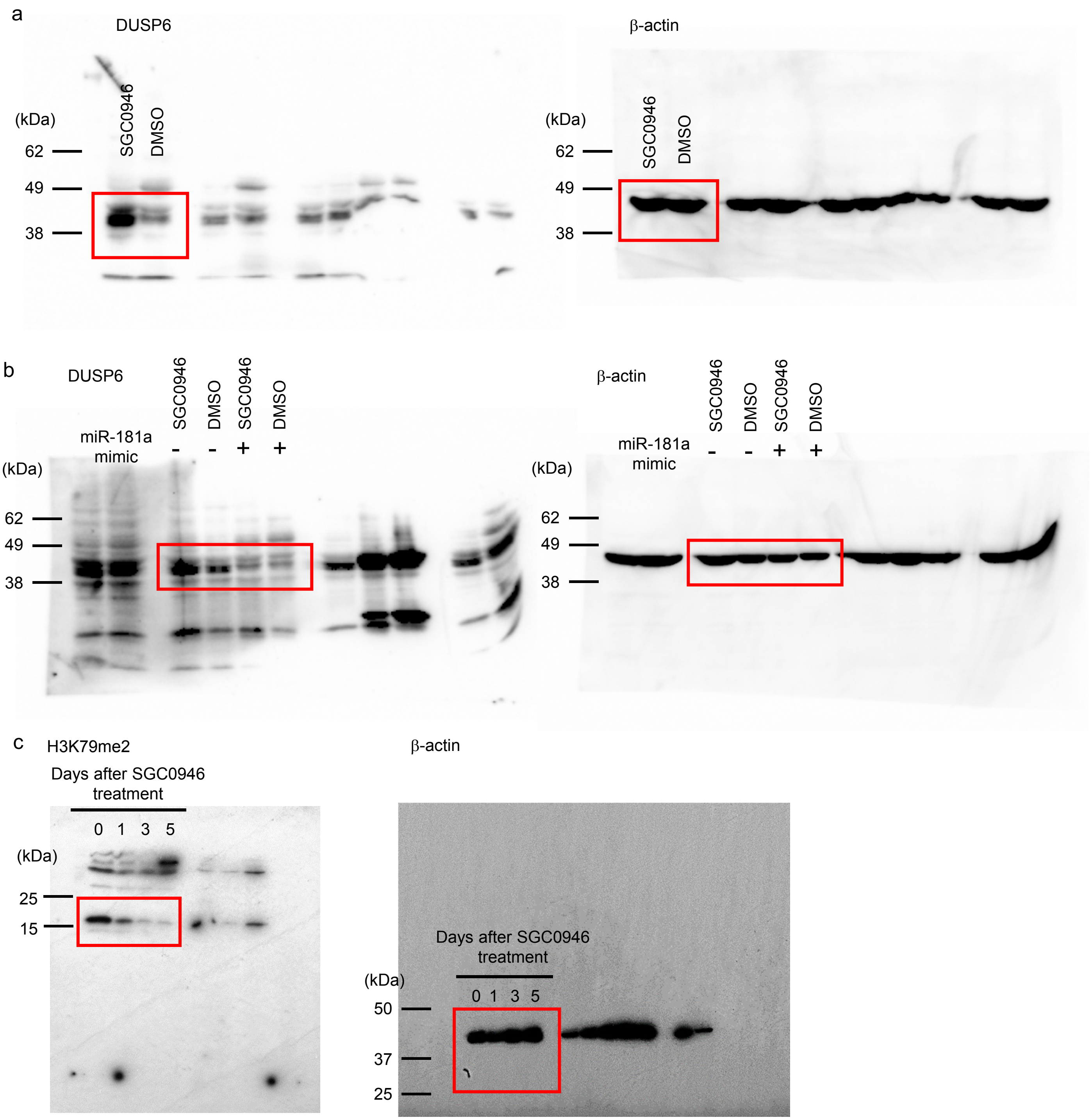

Supplementary Figure 31. Uncropped immunoblotting images from Figures 4d (a), 4k (b) and Supplementary Figure 4 (c). The red boxes indicate the specific bands presented in each Figure.

Supplementary Figure 32

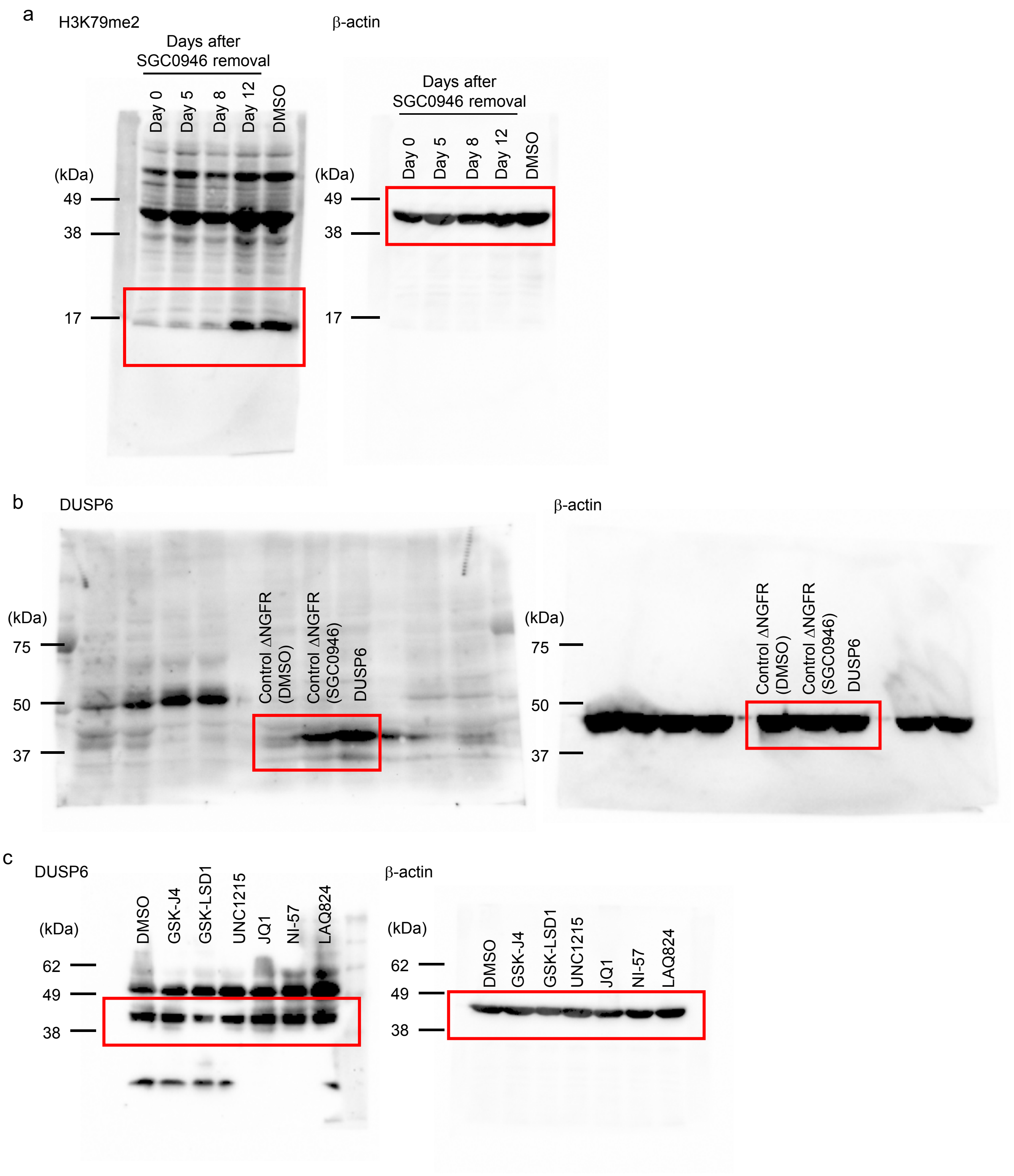

Supplementary Figure 32. Uncropped immunoblotting images from Supplementary Figures 9a (a), 14a (b) and 15a (c). The red boxes indicate the specific bands presented in each Figure.

Supplementary Figure 33

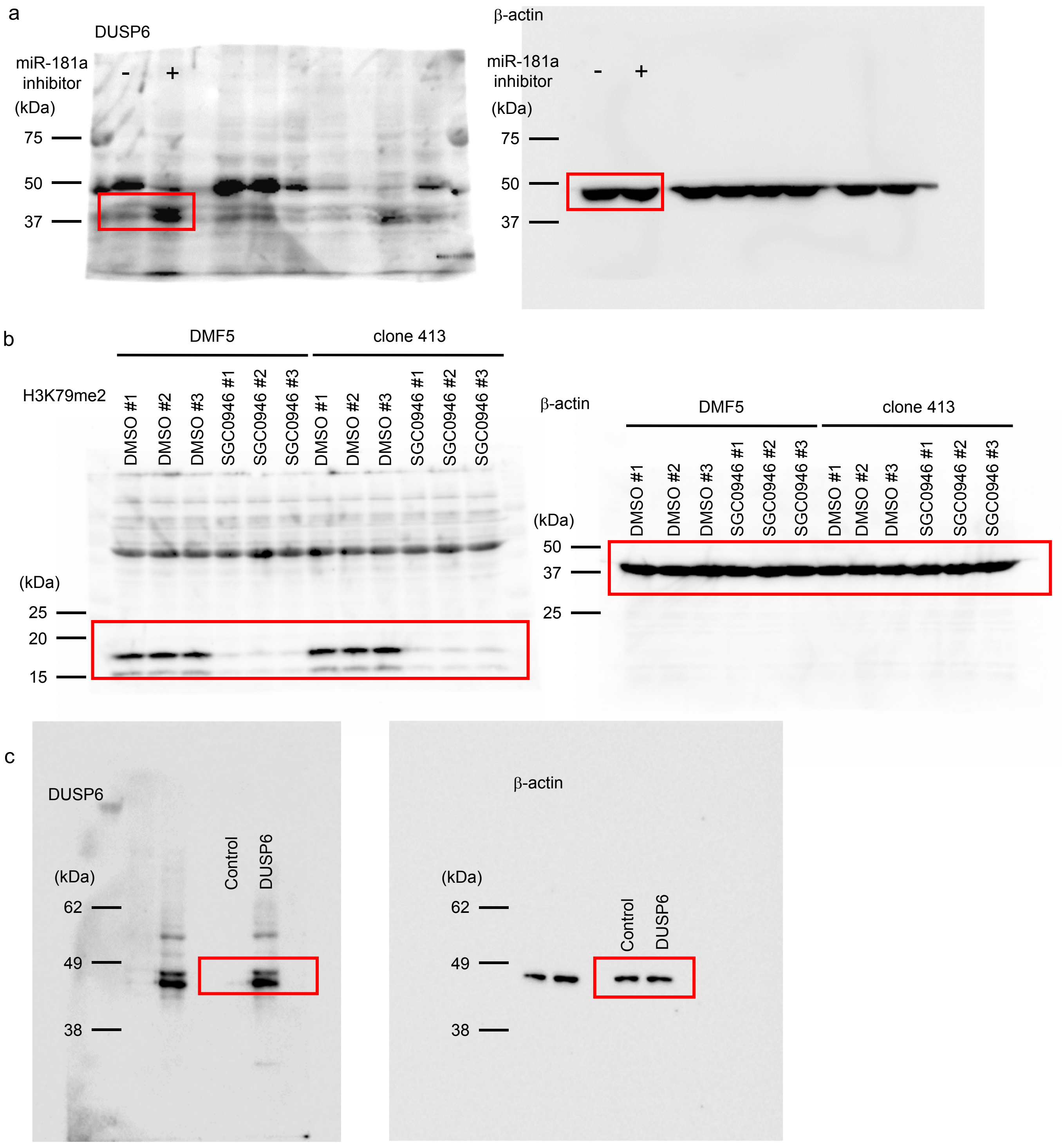

Supplementary Figure 33. Uncropped immunoblotting images from Supplementary Figures 16a (a), 18 (b) and 28b (c). The red boxes indicate the specific bands presented in each Figure.

Probe	Target	Dose (μM)	Ref
Histone-modifying enzymes			
GSK343	EZH2	0.5	1
GSK-J4	JMJD3/UTX	2	2
OICR-9429	WDR5	1	3
PFI-2	SETD7	5	4
GSK-LSD1	LSD1	1	5
SGC0946	DOT1L	0.5	6
LAQ824	HDAC	0.05	7
Histone readers			
JQ1	BET Bromodomain	0.15	8
GSK2801	BAZ2A/B	1	9
BAZ2-ICR	BAZ2A/B	1	10

Probe	Target	Dose (μM)	Ref
OF-1	BRPF1-3	1	
NI-57	BRPF1-3	0.5	
PFI-4	BRPF1B	0.5	
PFI-3	SMARCA4	1	11
I-BRD9	BRD9	1	12
UNC1215	L3MBTL3	1	13
SGC-CBP30	CREBBP/EP300	0.2	14
Other Targets			
LLY-507	SMYD2	1	15
GSK484	PAD4	1	16
Decitabine	DNMT	0.05	

Supplementary Table 1. Chemical probes specifically targeting epigenetic enzymes and effector molecules.

BB-z	TTTPAPRPPTPAPTIASQPLSLRPEACRPAAGGAVHTRGLDFACDIYIWAPLAGTCGVLLLSLVITLYCKRGRKKLLYIFKQPFMRPVQTTQEEDGCSCRFPEEEEGGCELPRVKFSRSADAPAYQQGQNQLYNELNLGRREEYDVLDKRRGRDPEMGGKPRRKNPQEGLYNELQKDKMAEAYSEIGMKGERRRGKGHDGLYQGLSTATKDTYDALHMQALPPR	Black: CD8α<br>Red: 4-1BB<br>Black: CD3z
28-BB-z	IEVMYPPPYLDNEKSNGTIIHVKGKHLCPSPLFPGPSKPFWVLVVVGGVLACYSLLVTVAFIIFWVRSKRSRLLHSDYMNMTPRRPGPTRKHYQPYAPPRDFAAYRSKRGRKKLLYIFKQPFMRPVQTTQEEDGCSCRFPEEEEGGCELPRVKFSRSADAPAYQQGQNQLYNELNLGRREEYDVLDKRRGRDPEMGGKPRRKNPQEGLYNELQKDKMAEAYSEIGMKGERRRGKGHDGLYQGLSTATKDTYDALHMQALPPR	Blue: CD28<br>Red: 4-1BB<br>Black: CD3z

Supplementary Table 2. Amino acid sequencing of the CD19-targeting CAR signaling domains used in this study.

Supplementary References

1. Verma SK*, et al.* Identification of Potent, Selective, Cell-Active Inhibitors of the Histone Lysine Methyltransferase EZH2. *ACS Med Chem Lett* **3**, 1091-1096 (2012).

2. Kruidenier L*, et al.* A selective jumonji H3K27 demethylase inhibitor modulates the proinflammatory macrophage response. *Nature* **488**, 404-408 (2012).

3. Grebien F*, et al.* Pharmacological targeting of the Wdr5-MLL interaction in C/EBPalpha N-terminal leukemia. *Nat Chem Biol* **11**, 571-578 (2015).

4. Barsyte-Lovejoy D*, et al.* (R)-PFI-2 is a potent and selective inhibitor of SETD7 methyltransferase activity in cells. *Proc Natl Acad Sci U S A* **111**, 12853-12858 (2014).

5. Mohammad HP*, et al.* A DNA Hypomethylation Signature Predicts Antitumor Activity of LSD1 Inhibitors in SCLC. *Cancer Cell* **28**, 57-69 (2015).

6. Yu W*, et al.* Catalytic site remodelling of the DOT1L methyltransferase by selective inhibitors. *Nat Commun* **3**, 1288 (2012).

7. Catley L*, et al.* NVP-LAQ824 is a potent novel histone deacetylase inhibitor with significant activity against multiple myeloma. *Blood* **102**, 2615-2622 (2003).

8. Filippakopoulos P*, et al.* Selective inhibition of BET bromodomains. *Nature* **468**, 1067-1073 (2010).

9. Chen P*, et al.* Discovery and Characterization of GSK2801, a Selective Chemical Probe for the Bromodomains BAZ2A and BAZ2B. *J Med Chem*, **59**, 1410-1424 (2015).

10. Drouin L*, et al.* Structure enabled design of BAZ2-ICR, a chemical probe targeting the bromodomains of BAZ2A and BAZ2B. *J Med Chem* **58**, 2553-2559 (2015).

11. Vangamudi B*, et al.* The SMARCA2/4 ATPase Domain Surpasses the Bromodomain as a Drug Target in SWI/SNF-Mutant Cancers: Insights from cDNA Rescue and PFI-3 Inhibitor Studies. *Cancer Res* **75**, 3865-3878 (2015).

12. Theodoulou NH*, et al.* Discovery of I-BRD9, a Selective Cell Active Chemical Probe for Bromodomain Containing Protein 9 Inhibition. *J Med Chem* **59**, 1425-1439 (2016).

13. James LI*, et al.* Discovery of a chemical probe for the L3MBTL3 methyllysine reader domain. *Nat Chem Biol* **9**, 184-191 (2013).

14. Hay DA*, et al.* Discovery and optimization of small-molecule ligands for the CBP/p300 bromodomains. *J Am Chem Soc* **136**, 9308-9319 (2014).

15. Nguyen H*, et al.* LLY-507, a Cell-active, Potent, and Selective Inhibitor of Protein-lysine Methyltransferase SMYD2. *J Biol Chem* **290**, 13641-13653 (2015).

16. Lewis HD*, et al.* Inhibition of PAD4 activity is sufficient to disrupt mouse and human NET formation. *Nat Chem Biol* **11**, 189-191 (2015).
