# Supplementary material for: DOT1L inhibition attenuates graft-versus-host disease by allogeneic T cells in adoptive immunotherapy models
Source: Nat Commun. 2018 May 15;9:1915. doi: 10.1038/s41467-018-04262-0 (PMC5954061; doi:10.1038/s41467-018-04262-0)
Supplement: Supplementary file 3 — Description of Additional Supplementary Files [file 41467_2018_4262_MOESM3_ESM.pdf]

Description of Additional Supplementary Files

File Name: Supplementary Data 1
Description: RNA sequencing analysis of differentially expressed genes between SGC0946- and DMSO-treated CD8+ T cells. Genes with P values <0.01 (paired two-sided t-test), FDR <0.1 and fold change of FPKM values >1.5 or <0.66 are shown.

File Name: Supplementary Data 2
Description: Differentially expressed genes following T cell stimulation retrieved from the publicly available data (GSE13887, paired two-sided t-test).
